# Sequencing and comparative genomic analysis of 1227 *Felis catus *cDNA sequences enriched for developmental, clinical and nutritional phenotypes

**DOI:** 10.1186/1471-2164-13-31

**Published:** 2012-01-18

**Authors:** Kristopher J Irizarry, Sukhaswami B Malladi, Xiangming Gao, Katherine Mitsouras, Lynda Melendez, Patricia A Burris, Jeffrey A Brockman, Samer W Al-Murrani

**Affiliations:** 1 College of Veterinary Medicine, Western University of Health Sciences, 309 East Second Street Pomona California, 91766, USA; 2 The Applied Genomics Center, Graduate College of Biomedical Sciences, Western University of Health Sciences, Pomona California 91766, USA; 3 Pet Hill's Pet Nutrition, Pet Nutrition Center, 1035 NE 43rd Street Topeka, KS 66617, USA; 4 College of Osteopathic Medicine of the Pacific, Western University of Health Sciences, Pomona California 91766, USA

**Keywords:** Feline, bioinformatics, comparative genomics, cDNA, annotation, gene ontology, OMIM, ortholog, nutrition, phenotype

## Abstract

**Background:**

The feline genome is valuable to the veterinary and model organism genomics communities because the cat is an obligate carnivore and a model for endangered felids. The initial public release of the Felis catus genome assembly provided a framework for investigating the genomic basis of feline biology. However, the entire set of protein coding genes has not been elucidated.

**Results:**

We identified and characterized 1227 protein coding feline sequences, of which 913 map to public sequences and 314 are novel. These sequences have been deposited into NCBI's genbank database and complement public genomic resources by providing additional protein coding sequences that fill in some of the gaps in the feline genome assembly. Through functional and comparative genomic analyses, we gained an understanding of the role of these sequences in feline development, nutrition and health. Specifically, we identified 104 orthologs of human genes associated with Mendelian disorders. We detected negative selection within sequences with gene ontology annotations associated with intracellular trafficking, cytoskeleton and muscle functions. We detected relatively less negative selection on protein sequences encoding extracellular networks, apoptotic pathways and mitochondrial gene ontology annotations. Additionally, we characterized feline cDNA sequences that have mouse orthologs associated with clinical, nutritional and developmental phenotypes. Together, this analysis provides an overview of the value of our cDNA sequences and enhances our understanding of how the feline genome is similar to, and different from other mammalian genomes.

**Conclusions:**

The cDNA sequences reported here expand existing feline genomic resources by providing high-quality sequences annotated with comparative genomic information providing functional, clinical, nutritional and orthologous gene information.

## Background

The domestic cat, *Felis catus*, is a member of the family Felidae and represents the *Feliformia *branch of the order Carnivora [1]. The domestic cat is an important companion animal and veterinary species. There are roughly 82 million companion cats living in more than 35 million US households [2]. The domestic cat also has substantial value as a model organism for comparative mammalian genomics because it is an obligate carnivore [3], unlike the dog which is an omnivore [4]. Additionally, the domestic cat is an important model organism for Felidae because of its close phylogenetic relationship to the wildcat (*Felis silvestris)*, the sand cat (*Felis margarita*), the black-footed cat (*Felis nigripes*) and the jungle cat (*Felis chaus*). It can also serve as a model for more distantly related felid species including pumas such as the Cheetah (*Acinonyx jubatus*), lynx species, ocelots [5,6], and members of panthera including the lion (*Panthera leo*), the tiger (*Panthera tigris*), and snow leopard (*Uncia uncia*) [7]. A major goal of feline genomics is to identify and decode both cat-specific biology as well as conserved mammalian biology. The identification of feline-specific biochemistry and physiology is required in order to better understand the unique nutritional and veterinary needs of cats and to enhance the wellness of domestic cats as well as the health and management of captive felid species.

A number of cat-specific biological adaptations have been described to date. Cats exhibit a variety of evolutionary adaptations thought to be associated with their predatory behaviour and obligate carnivore status. For example, domestic cats exhibit distinct distal forelimb anatomical adaptations associated with predation [8,9], as well as sensory adaptations in both sound perception [10-12] and visual acuity [13,14]. At a molecular level, cats exhibit differences in the regulation of sugar transporters [15] resulting in lower liver glucose transporter activity [16] and differences in carbohydrate metabolism compared to omnivores [17]. Because the carnivore diet is relatively high in amino acid content, adult cats maintain blood glucose levels from gluconeogenesis of glucogenic amino acids, lactic acid and glycerol [18]. Compared to omnivorous mammals, in which gluconeogenesis occurs in the post absorptive state, cats exhibit the greatest extent of gluconeogenesis right after a meal during the absorptive state [19].

Amino acid biosynthesis and deficiency has been relatively well studied in domestic cats. Cats have dietary requirements for the amino acids taurine [20], arginine [21], cysteine and, methionine [22]. Arginine deficiency in cats has been associated with rapid onset of hyperammonemia characterized by severe signs of ammonia toxicity [23]. The sulphur containing amino acids cysteine and methionine are normally present in high amounts in animal flesh and are required for normal feline development [24,25]. The beta-amino sulfonic acid taurine is required in cats because, unlike many other species which can conjugate bile acids to either glycine or taurine for secretion of bile salts into bile, cats can only use taurine. Unlike dogs, cats have evolved limited capacity to synthesize taurine [26], subsequently, taurine deficiency in cats is associated with abnormal cardiac [27], immune [28], neurological [29], platelet [30], reproductive [31] and retinal [32] dysfunctions. The recent description of the taurine transporter knock out mouse underscores the biological roles of taurine in mammals [33].

Although many aspects of feline-specific biology have been elucidated to date, bioinformatics methods and comparative genomics approaches can provide a mechanism for producing a number of plausible and useful biological hypotheses from feline cDNA sequences.

The 2007 release of the feline genome [34] marked the beginning of the feline genomics era, which was followed by the identification of close to 1 million single nucleotide polymorphisms across cat breeds [35] which further extends the repertoire of genomic tools for investigating the genomic basis of feline phenotypes. In this paper, we describe the sequencing of additional feline cDNA sequences and demonstrate the utility of employing comparative genomics methods to investigate, not only the roles of these cDNA sequences, but the extent to which these feline sequences diverge from other mammalian orthologous sequences.

Our working hypothesis is that conservation among human, mouse, dog and cat orthologs underscores conserved mammalian biology while feline sequence divergence among mammalian orthologs provides potential insight into cat-specific biology. Specifically, we employ a computational comparative gene expression analysis to map the cDNA sequences to anatomical information, developmental timelines, cells and pathology terms. Additionally, we utilize the gene ontology annotation, in combination with measures of synonymous and non-synonymous differences in orthologous protein sequences, to better understand which of the cDNA sequences are likely to represent conserved mammalian biology and which are more likely to represent feline-specific biology. We organize these results into biological processes, cellular localization and molecular function in order to more easily interpret the results. Finally, we map these feline cDNA sequences to orthologs in other species in order to identify (1) phenotypes, (2) biochemical pathways and (3) human diseases in an attempt to better understand the roles of these cDNA sequences in feline development, nutrition and disease.

## Results

### Sequencing and Orthologue Identification

**Figure 1:**
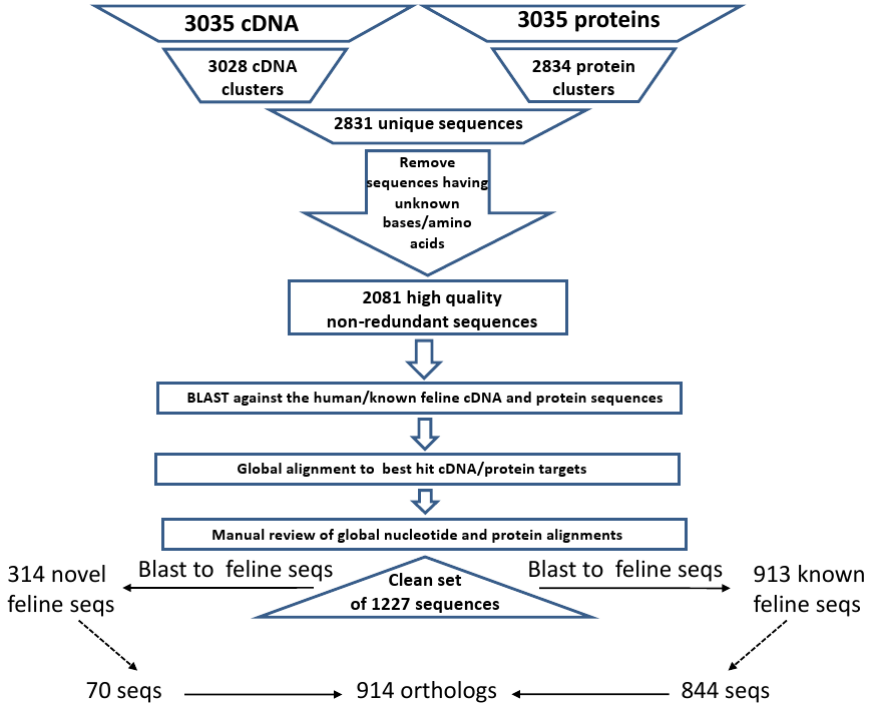
**Schematic Representation of Identification of 1227 Feline cDNA Sequences**. An initial set of 3035 cDNA sequences were clustered in nucleotide and protein space to identify the longest representative sequence for each cluster. The intersection of the set of cDNA and protein clusters resulted in a set of 2831 cDNA sequence clusters. All sequences within this set that contained N's were removed resulting in a set of 2081 high quality, non-redundant cDNA sequences. These sequences were blasted against the (1) set of ensembl human known cDNA and protein sequences and (2) feline known cDNA and protein sequences. Global alignments were generated for each cDNA blast hit and manually inspected for quality. The final set of 1227 cDNA sequences corresponded to 913 known feline cDNA sequences and 314 novel feline sequences. Blasting to dog, human and mouse sequences identified a total of 914 orthologs, corresponding to 70 novel and 844 known sequences.

1227 high quality feline cDNA sequences were identified from a starting set of 3035 cDNA sequences (Figure 1). Total RNA was purified from 21 feline tissues (brain, kidney medulla/cortex, spleen, heart, liver, lung, skeletal muscle, thyroid gland, lymph node, pancreas, adrenal gland, tongue, colon, mammary gland, neonatal thymus, brain and testes) collected from 10 domestic short-haired cats post-mortem, three cell lines derived from kidney, brain, lung, and 1 tissue pool using standard procedures. The initial set of 3035 cDNA sequences was assembled from the sequencing reads from tissue specific cDNA libraries. These sequences were designated full length because they corresponded to the complete length of assembled sequencing reads. These sequences were translated to produce protein sequences and clustered in nucleotide space and protein space to identify a set of non-redundant full length sequences. The results of the clustering produced 3028 nucleotide clusters and 2834 protein clusters. The intersection of these two sequence sets was used to produce the final clustered full length sequences, for which there were 2831 sequences. The set of clustered sequences were filtered to remove sequences containing non-nucleotide and non-amino acid letters which resulted in a set of 2081 high quality non-redundant full length sequences.

For the set of 2081 cDNA sequences, the shortest and longest sequences were 353 and 4750 nucleotides respectively. The average nucleotide length was 1349 nucleotides with a standard deviation of 567 nucleotides. The 2081 protein sequence set exhibited a shortest and longest sequence of 41 and 1128 amino acids respectively. The average protein sequence length was 279 amino acids with a standard deviation of 149 amino acids.

This set of sequences was used to blast against the set of known human cDNA and protein sequences to identify the best human match (see Figure 1). Additionally, these 2081 cDNA sequences were blasted against known and *ab initio *feline cDNA and protein sequences from ensemble [36] to identify sequences for which public feline sequence data exists. Subsequently, these sequences were aligned using a global alignment algorithm to remove sequences for which the best blast hit represented only local homology. After manual review of all of the global nucleotide and protein alignments, a set of 1227 non-redundant feline sequences were selected as high confidence, high quality feline sequences. Within the set of 1227 sequences, 913 *known *sequences and 314 *novel *sequences were identified for which 914 were successfully mapped to their corresponding dog, human and mouse orthologs. Although additional non-redundant feline cDNA sequences we identified mapped to three or fewer orthologs across the four species, we limited our subsequent analysis to only those sequences for which all three non-feline species orthologs were confidently identified. This decision was made to ensure that our functional and comparative analysis would include only feline cDNA sequences for which dog, mouse and human orthologs were identified. Of the 914 orthologous sequence set, 844 sequences corresponded to *known *feline sequences and 70 corresponded to novel sequences (see Figure 1). Additional file 1, Table S1 contains the complete set of 1227 non-redundant nucleotide and protein sequences. The complete set of 914 orthologous sequences is listed in Additional file 2, Table S2 along with the designation of *known *or *novel *and the corresponding ensembl gene, transcript and protein identifiers for the dog, human and mouse orthologs.

**Figure 2:**
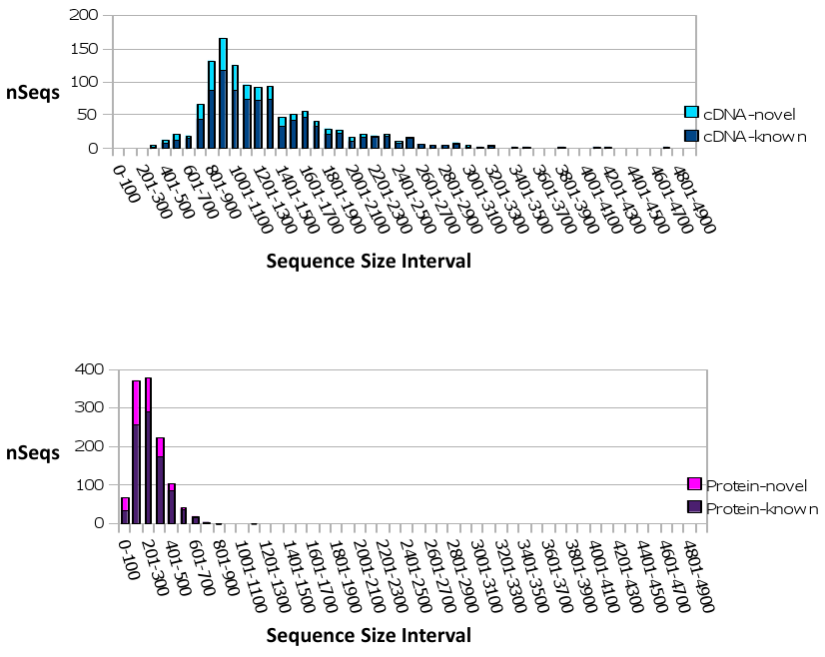
**Distribution of 1227 cDNA and Protein Sequences by Size**. The set of 1227 sequences were assessed for their size distribution. The distribution of all 1227 cDNA sequences is shown on the top panel with dark blue (lower) denoting the subset of known cDNA sequences and light blue (upper) denoting the subset of novel cDNA sequences. The distribution of all 1227 protein sequences is shown on the bottom panel with purple (lower) representing the subset of known protein sequences and magenta (upper) indicating the subset of novel protein sequences. Sequence counts are by interval of size 100.

It is interesting to note that compared to the existing public feline sequences, the sequences we identified exhibited a trend toward longer length and fewer sequencing errors. For example, of the 913 sequences that correspond to known feline public sequences, 309 of the public sequences contain a non-nucleotide sequence character such as an N or an X. Within those public sequences containing N's or X's, 292 are shorter than the corresponding sequence we identified and only 17 of the public sequences containing non nucleotide letters are longer than the sequences we identified. Within the set of 604 public sequences mapped to our known sequences that do not contain N's or X's, 597 public feline sequences are shorter in length than the feline sequence we identified with only 7 public sequences having a longer length than our feline sequences. Figure 2 shows the distribution of nucleotide and protein sequence lengths for our set of 1227 sequences.

### Comparative Gene Expression Analysis

The sequences we report were obtained from extensive sequencing of 21 individual tissue cDNA libraries and 1 pooled cDNA library. It is well known that while some genes may exhibit rather narrow ranges of expression across tissues and cell types, many genes exhibit expression across numerous tissues and cell types [37,38]. We chose to leverage the orthologous relationships among our sequences to infer gene expression patterns across a set of anatomical regions.

When considering the inferred gene expression patterns as a function of anatomical regions, we were able to identify 114 anatomical regions exhibiting expression of 766 genes encoding our sequences. The range of gene counts, we identified, was from 1 gene each in lymph, rectum and cerebrum to 752 genes for the anatomical term lung. The eight anatomical terms exhibiting the lowest gene counts with more than a single gene include middle ear, corpus callosum and trachea (2 genes each, 0.21%), subthalamic nucleus and foreskin (3 genes each, 0.32%), epidermis and ciliary body (4 genes each, 0.44%) followed by adrenal medulla and internal ear (5 genes each, 0.54%). The eight anatomical terms exhibiting the greatest gene counts, each contain at least 73% of the genes corresponding to our cDNA sequences. The top eight anatomical regions listed in ascending order are liver (668 genes, 73%), skin (676 genes, 74%), colon (686 genes, 75%), placenta (689 genes, 75%), kidney (693 genes, 76%), testis (703 genes, 77%), brain (725 genes, 79%) and lung (752 genes, 82%). Table 1 contains the anatomical gene expression annotation results.

**Table 1:** Inferred Anatomical Gene Expression Patterns

*Anatomical Region*	*Number of Genes*	*% of Genes*
adrenal cortex	206	22.5383

adrenal gland	195	21.3348

adrenal medulla	5	0.547

alveolus	164	17.9431

amnion	27	2.954

amniotic fluid	61	6.674

amygdala	39	4.267

aorta	234	25.6018

artery	53	5.7987

atrium	13	1.4223

bile duct	247	27.0241

bladder	365	39.9344

blood	546	59.7374

bone	598	65.4267

bone marrow	399	43.6543

brain	725	79.3217

breast	474	51.86

cartilage	494	54.0481

cerebellum	121	13.2385

cerebellum cortex	43	4.7046

cerebral cortex	85	9.2998

cerebrum	1	0.1094

cervix	443	48.4683

choroid	469	51.3129

ciliary body	4	0.4376

cochlea	158	17.2867

colon	686	75.0547

cornea	149	16.302

corpus callosum	2	0.2188

developmental	34	3.7199

duodenum	138	15.0985

dura mater	58	6.3457

endometrium	518	56.674

epidermis	4	0.4376

epididymis	92	10.0656

foreskin	3	0.3282

fovea centralis	434	47.4836

frontal lobe	356	38.9497

gall bladder	70	7.6586

ganglion	9	0.9847

germinal center	438	47.9212

greater omentum	16	1.7505

gum	21	2.2976

head and neck	428	46.8271

heart	629	68.8184

hippocampus	301	32.9322

hypopharynx	102	11.1597

hypothalamus	311	34.0263

internal ear	5	0.547

intestine	313	34.2451

iris	138	15.0985

islets of Langerhans	557	60.9409

kidney	693	75.8206

lacrimal gland	69	7.5492

larynx	239	26.1488

lens	475	51.9694

liver	668	73.0853

lung	752	82.2757

lymph	1	0.1094

lymph node	426	46.6083

lymphoreticular	164	17.9431

macula lutea	434	47.4836

mammary gland	544	59.5186

medulla oblongata	144	15.7549

meninges	57	6.2363

mesenchyma	36	3.9387

middle ear	2	0.2188

muscle	242	26.477

myocardium	130	14.2232

nasopharynx	240	26.2582

nervous	9	0.9847

oesophagus	85	9.2998

optic nerve	445	48.6871

oral cavity	21	2.2976

ovary	660	72.2101

pancreas	568	62.1444

parathyroid	490	53.6105

peripheral nerve	104	11.3786

pharynx	336	36.7615

pia mater	57	6.2363

pineal body	65	7.1116

pineal gland	120	13.1291

pituitary gland	218	23.8512

placenta	689	75.3829

prostate	651	71.2254

rectum	1	0.1094

retina	568	62.1444

salivary gland	386	42.2319

skeletal muscle	503	55.0328

skin	676	73.9606

small intestine	28	3.0635

smooth muscle	134	14.6608

spinal cord	36	3.9387

spinal ganglion	122	13.3479

spleen	571	62.4726

stomach	641	70.1313

substantia nigra	13	1.4223

subthalamic nucleus	3	0.3282

sympathetic chain	121	13.2385

synovium	69	7.5492

testis	703	76.9147

thymus	168	18.3807

thyroid	430	47.046

tongue	164	17.9431

tonsil	144	15.7549

trabecular meshwork	100	10.9409

trachea	2	0.2188

trophoblast	70	7.6586

umbilical cord	53	5.7987

urinary	108	11.8162

uterus	661	72.3195

vein	135	14.7702

visual apparatus	583	63.7856

whole body	480	52.5164

The anatomical expression pattern of the gene corresponding to each cDNA sequence was inferred. The human orthologs of each cDNA sequence were used to infer anatomical gene expression patterns using expression data (egenetic data) obtained from biomart. The results include 114 anatomical regions exhibiting expression of 766 genes encoding the cDNA sequences. The number of genes with inferred expression in each region is indicated (Number of Genes), as well the percentage of genes with inferred expression in each region (% of Genes).

The expression pattern annotation corresponding to cell type resulted in gene counts for 44 cell types ranging from 1 gene (0.1%) each for brown adipose cell, platelet and eosinophil to 2 genes (0.22%) in mast cell and 3 genes (0.33%) in hepatocyte. A count of 13 genes (1.4%) was obtained for monocytes, while counts of 33 genes (3.7%) each were reported for both cardiac muscle cell and chondrocyte. At the other end of the expression spectrum, the term stem cell was associated with 626 genes (70.8%), B-lymphocyte (628 genes, 70.7%), epithelium (604 genes, 68.5%), retinal pigment epithelium (514 genes, 57%), skeletal muscle cell (499 genes, 56.6%), fibroblast (485 genes, 55%) and germ cell (435 genes, 49%). The cell expression counts provide cellular expression annotation for 749 of our orthologous genes. Table 2 contains the counts for all of the cell type expression annotations.

**Table 2:** Inferred Cell Type Gene Expression Patterns

*Cell Type*	*Number of Genes*	*% of Genes*
adipocyte	188	21.23

alveolar macrophage	127	14.11

B-lymphoblast	106	12.58

B-lymphocyte	628	70.79

brown adipose	1	0.11

cardiac muscle cell	33	3.72

chondrocyte	33	3.72

dendritic cell	56	6.35

endothelium	194	21.88

eosinophil	1	0.11

epithelium	604	68.49

fibroblast	485	54.92

foam cell	25	2.74

germ cell	435	49.02

glial cell	432	48.69

glioblast	42	4.7

granulosa cell	63	7.22

hepatocyte	3	0.33

keratinocyte	287	32.17

leukocyte	295	33.26

lymphocyte	86	9.63

macrophage	194	21.88

mast cell	2	0.22

melanocyte	283	31.84

monocyte	13	1.42

muscle cell	233	26.37

myeloid cell	149	17.4

natural killer cell	339	38.84

neuroblast	396	44.86

neuroepithelium	143	16.19

neuron	124	14

pericyte	128	14.66

platelet	1	0.11

proerythroblast	65	7.77

promyeloblast	14	1.86

promyelocyte	44	5.03

retinal pigment epithelium	514	57.55

skeletal muscle cell	499	56.56

smooth muscle cell	128	14.77

squamous cell	376	42.67

stem cell	626	70.79

T-lymphocyte	359	40.92

transitional	194	22.21

white adipose	41	4.81

The cell type expression pattern of the gene corresponding to each cDNA sequence was inferred. The human orthologs of each cDNA sequence were used to infer cell type gene expression patterns using expression data (egenetic data) obtained from biomart. The results include expression across 44 cell types. The number of genes with inferred expression in each cell type is indicated (Number of Genes), as well the percentage of genes with inferred expression in each cell type (% of Genes).

The mapping of pathology term expression annotation with our orthologous gene sets resulted in 57 terms having gene counts. The terms with the fewest gene counts included ulcerative colitis, neoplasia, rheumatoid arthritis, cirrhosis, and hyperplasia each of which exhibited a count of 1 gene (0.1%). These terms were immediately followed by choriocarcinoma (6 genes, 0.66%), seminoma (7 genes, 0.77%), carcinoma in situ (10 genes, 1.1%), liposarcoma (12 genes, 1.3%) and schwannoma (13 genes, 1.5%). The pathological terms with the greatest counts include normal (765 genes, 86.4%), carcinoma (715 genes, 81%), adenocarcinoma (684 genes, 77.5%), tumor (633 genes, 72%), chondrosarcoma (580 genes, 65%) and glioblastoma (508 genes, 57%). A total of 766 genes were annotated with pathological terms for gene expression. Table 3 indicates the annotated gene count corresponding to inferred gene expression for each of the pathological terms.

**Table 3:** Inferred Pathology Gene Expression Patterns

*Pathology*	*Number of Genes*	*% of Genes*
ulcerative colitis	1	0.1094

neoplasia	1	0.1094

rheumatoid arthritis	1	0.1094

cirrhosis	1	0.1094

hyperplasia	1	0.1094

choriocarcinoma	6	0.6565

seminoma	7	0.7659

carcinoma in situ	10	1.0941

liposarcoma	12	1.3129

Schwannoma	13	1.5317

arthritis	15	1.86

goitre	21	2.6258

papillary serous carcinoma	27	2.954

phaeochromocytoma	29	3.3917

monocytic	32	3.5011

schizophrenia	59	6.7834

sarcoma	62	6.8928

Denys-drash	70	8.2057

Wilms	76	8.4245

fibrosarcoma	95	10.7221

Ewing's	101	11.5974

osteosarcoma	110	12.4726

lymphoblastic	106	12.5821

hypertrophic cardiomyopathy	126	14.2232

medulloblastoma	141	15.6455

osteoarthritis	145	16.0832

fibrothecoma	150	17.3961

Burkitt's	161	18.8184

lymphocytic	180	20.2407

myeloma	195	22.3195

glioma	226	25.4923

enchondroma	234	26.2582

rhabdomyosarcoma	233	26.477

leukaemia	234	26.9147

teratocarcinoma	247	28.1182

myeloid	247	28.2276

malignant tumour	280	31.291

astrocytoma	281	31.6193

insulinoma	281	31.9475

retinoblastoma	297	34.0263

cystic fibrosis	302	34.1357

lymphoma	309	35.3392

T-cell leukemia	317	36.3239

adenoma	345	38.2932

meningioma	345	38.9497

leiomyosarcoma	383	43.5449

carcinoid	431	48.3589

ascites	428	48.5777

neuroblastoma	451	51.0941

oligodendroglioma	463	52.1882

melanoma	475	54.0481

glioblastoma	508	57.3304

chondrosarcoma	580	65.4267

tumour	633	71.663

adenocarcinoma	684	77.5711

carcinoma	715	80.9628

normal	765	86.4333

The pathology expression pattern of the gene corresponding to each cDNA sequence was inferred. The human orthologs of each cDNA sequence were used to infer pathology gene expression patterns using expression data (egenetic data) obtained from biomart. The results include 57 pathology terms exhibiting expression of the cDNA sequences. The number of genes with inferred expression in each type of pathology is indicated (Number of Genes), as well the percentage of genes with inferred expression in each type of pathology (% of Genes).

Finally, the gene expression annotation associated with developmental stages offers some insight into the overall timing of gene expression across an organism's life time. The results suggest that the greatest numbers of genes are associated with the developmental stage terms of fetus (724 genes, 82%) and embryo (635 genes, 72%) while the fewest genes are associated with developmental stage terms child (76 genes, 8.4%) and adolescent (61 genes, 6.7%). Additional developmental stage terms include specific weeks of gestation, such as week 4 (30 genes, 3.2%), week 6 (50 genes, 5.6%), week 8 (318 genes, 36.4%) as well as stages towards the end of gestation, such as week 26 (193 genes, 22%), week 32 (97 genes, 11%) and week 42 (314 genes, 35%). Other developmental terms include stages indicated by years, such as 2 years (316 genes, 36%), 3 years (173 genes, 20%), 14 years (181 genes, 20%), 21 years (53 genes, 6%), 45 years (150 genes, 17%) and 89 years (133 genes, 15%). The results are shown in Table 4.

**Table 4:** Inferred Developmental Gene Expression Patterns

*Developmental Stage*	*Number of Genes*	*% of Genes*
embryo	635	71.9912

fetus	724	81.8381

infant	211	23.8512

child	76	8.4245

adolescent	61	6.7834

adult	753	85.1204

4 weeks	30	3.2823

6 weeks	50	5.5799

7 weeks	3	0.3282

8 weeks	318	36.4333

9 weeks	562	63.7856

10 weeks	253	29.3217

11 weeks	32	3.8293

12 weeks	367	41.5755

14 weeks	26	2.8446

15 weeks	55	6.4551

16 weeks	326	35.9956

17 weeks	179	19.6937

18 weeks	225	25.0547

19 weeks	594	67.1772

20 weeks	602	67.8337

21 weeks	307	33.9168

22 weeks	316	35.0109

24 weeks	324	36.5427

26 weeks	193	21.663

32 weeks	97	10.9409

42 weeks	314	35.3392

2 years	316	36.3239

3 years	173	19.8031

6 years	103	11.9256

14 years	181	20.4595

16 years	119	13.3479

17 years	35	3.9387

19 years	62	7.1116

20 years	49	5.5799

21 years	53	6.1269

23 years	193	21.7724

24 years	181	20.4595

25 years	308	34.7921

26 years	331	37.3085

27 years	277	31.1816

28 years	129	14.442

31 years	255	28.337

34 years	65	7.4398

35 years	22	2.6258

36 years	150	17.1772

40 years	1	0.1094

44 years	27	2.954

45 years	150	17.0678

46 years	352	40.0438

47 years	2	0.2188

48 years	1	0.1094

49 years	252	28.4464

55 years	221	24.9453

58 years	1	0.1094

60 years	1	0.1094

62 years	226	25.3829

64 years	152	17.2867

69 years	182	20.5689

70 years	283	31.7287

71 years	230	25.8206

72 years	131	14.9891

73 years	1	0.1094

74 years	12	1.3129

76 years	10	1.0941

79 years	1	0.1094

80 years	133	14.8796

89 years	14	1.6411

The developmental expression pattern of the gene corresponding to each cDNA sequence was inferred. The human orthologs of each cDNA sequence were used to infer developmental stage by week of gestation, year of age and life stage of human development. The number and percentage of genes with inferred expression in each stage is indicated in the second and third columns respectively.

Taken together these results suggest that the genes encoding the cDNA sequences we have identified exhibit considerably larger breadth of expression than would be suggested from the initial tissues that were sequenced. The broad extent of tissue, cell type, developmental and pathological expression annotation suggests that these sequences may include sequences underlying tissue and organ development as well as contributing to specific pathological conditions. In order to better understand the biological role of these genes we chose to combine the expression annotation with other functional and comparative annotation types.

### Gene Ontology Annotation Analysis

Gene ontology (GO) annotation was performed on the feline sequences using the previously identified comparative genomics ortholog relationships. Gene ontology terms were mapped from human annotation files to feline orthologs. The initial gene ontology human molecular function annotation file contained 73,467 function annotation terms mapped to 21,956 human gene identifiers, corresponding to 3,085 unique gene ontology function terms. The cellular location gene ontology annotation file contained 975 unique terms mapped to 21,956 human genes resulting in 69,556 gene-term relationships. The biological process gene ontology annotation also contained 21,956 human gene identifiers consisting of 6518 unique gene ontology process annotation terms represented by 89,968 gene-to-GO entries.

The mapping of gene ontology functional annotation terms onto the non-redundant full length sequences resulted in 901 of our feline cDNA sequences becoming associated with 647 unique gene ontology molecular function annotation terms resulting in 3219 annotation-gene relationships. Repeating the procedure to map the cellular location annotation, we mapped 3423 gene-annotation relationships corresponding to 337 unique location annotation terms covering the set of 901 genes. Mapping the biological process annotation terms produced 4247 gene-to-GO annotations of which 1441 unique gene ontology process annotations mapped successfully to 901 genes.

Typically gene ontology annotation terms are filtered using an enrichment criterion that is calculated from a hypergeometric null model to describe the number of annotation terms one might expect to occur within a gene set of a given size and a GO annotation distribution of particular parameters. Although such an approach is necessary when attempting to determine the biological role of a gene set, such as up-regulated or down-regulated genes in a gene expression study, we did not calculate an enrichment of gene ontology terms, instead we combined the gene ontology annotation with measures of evolutionary selection using non-synonymous (dN) versus synonymous (dS) codon statistics as a means of exploring the evolutionary relationships that exist among the different gene ontology annotations across our cDNA sequences. A well accepted approach for identifying evidence of positive selection is to identify genes exhibiting significantly larger rates of non-synonymous substitutions per non-synonymous site than synonymous substitutions per synonymous site. Evidence of fixation exists when the ratio of non-synonymous substitution rate to synonymous substitution rate equals zero (dN/dS = 0).

Evidence of negative selection exists when dN/dS < 1 and evidence of positive selection exists when dN/dS > 1. We recognize that using the dN/dS value across an entire gene is an extremely conservative measure of selection, and that smaller regions within a gene may exhibit local signals of positive selection [39]. However, we chose the conservative approach in order to minimize reporting false positives due to the possibility of sequencing errors.

Instead of considering all of the genes we identified as a single gene set, we chose to select gene subsets using SQL queries in MySQL to identify cDNA sequences sharing gene ontology annotation terms for which we calculated an average dN/dS value. From this analysis, we were able to identify annotation types exhibiting low dN/dS values, corresponding to greater levels of sequence conservation across species. We were also able to identify annotation terms that exhibited considerably higher dN/dS values indicating less negative selection in the act on some types of genes. Because we chose to employ a stringent criteria for positive selection, we did not identify genes exhibiting strong signals of positive selection, instead, we were able to identify genes and annotation types with different levels of selection pressure acting on them. Beginning with the gene ontology location annotation, an SQL query was performed such that the genes exhibiting the same location annotation terms were grouped together and the average dN/dS value was calculated for cat versus dog, cat versus human and cat versus mouse. Location annotations occurring within gene sets that exhibit extremely low dN/dS values and very low standard deviation of the dN/dS value for each species were selected as negatively selected location annotation gene sets.

**Figure 3:**
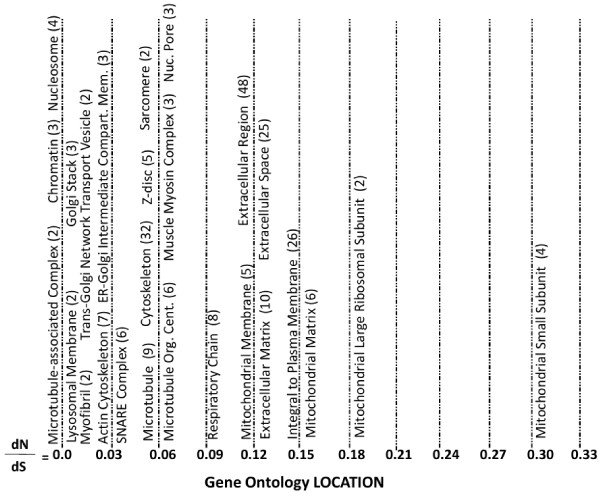
**Gene Ontology Location Terms by dN/dS Value**. Representative gene ontology location terms associated with the proteins encoded by the feline cDNA sequences were stratified by dN/dS values of cat versus dog, human and mouse. The number of feline cDNAs associated with each annotation term is indicated in parentheses.

A number of genes grouped by the same gene ontology location annotation terms exhibited dN/dS values close to zero, (dN/dS < 0.07). These genes were associated with several cellular themes which were each associated with multiple location annotation terms. See Figure 3 for a representative map of gene ontology location annotation terms across the dN/dS values. The following terms related to microtubules and cytoskeletal organization occurred: microtubule associated complex (2 genes, dN/dS = 0), actin cytoskeleton (7 genes, dN/dS = 0.03), microtubule (9 genes, dN/dS = 0.05), cytoskeleton (32 genes, dN/dS = 0.05) and microtubule organizing center (6 genes, dN/dS = 0.06). A muscle theme was present within the negatively selected location annotations. Muscle associated location terms included myofibril (2 genes, dN/dS = 0.02), Z disc (5 genes, dN/dS = 0.04), sarcomere (2 genes, dN/dS = 0.05) and muscle myosin complex (3 genes, dN/dS = 0.06). Additional location terms within this group included chromatin (3 genes, dN/dS = 0), nucleosome (4 genes, dN/dS = 0) and nuclear pore (3 genes, dN/dS = 0.06). The last theme observed within this group relates to intracellular trafficking and includes terms such as lysosomal membrane (2 genes, dN/dS = 0.01), golgi stack (3 genes, dN/dS = 0.01), trans golgi network transport vesicle (2 genes, dN/dS = 0.02), ER-Golgi intermediate compartment membrane (3 genes, dN/dS = 0.03) and SNARE complex (6 genes, dN/dS = 0.04).

Location annotation terms associated with genes exhibiting a larger dN/dS value included extracellular region (48 genes, dN/dS = 0.12), extracellular matrix (10 genes, dN/dS = 0.13), extracellular space (25 genes, dN/dS = 0.13) and integral to plasma membrane (26 genes, dN/dS = 0.14). Some of the location terms with the greatest dN/dS values were associated with the mitochondria, for example respiratory chain (8 genes, dN/dS = 0.1), mitochondrial membrane (5 genes, dN/dS = 0.12), mitochondrial matrix (6 genes, dN/dS = 0.16), mitochondrial large ribosomal subunit (2 genes, dN/dS = 0.19) and mitochondrial small subunit (4 genes, dN/dS = 0.31).

**Figure 4:**
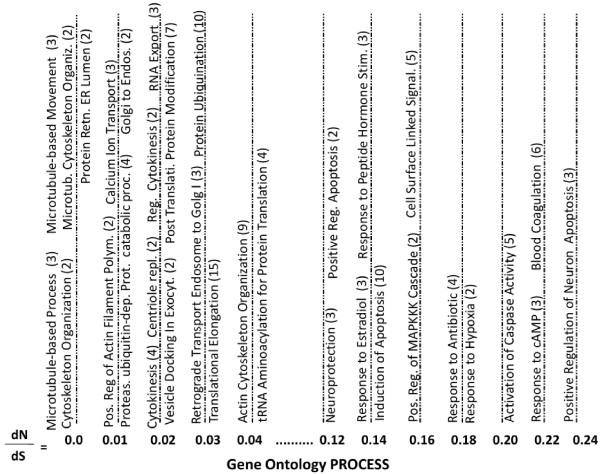
**Gene Ontology Process Terms by dN/dS Value**. A representative sample of gene ontology process terms associated with the proteins encoded by the feline cDNA sequences were stratified by dN/dS values of cat versus dog, human and mouse. The number of feline cDNAs associated with each annotation term is indicated in parentheses.

Similar themes were observed within the gene ontology process annotation data. See Figure 4 for a representative map of gene ontology process annotation by dN/dS values. The microtubule theme was represented by the terms microtubule-based process (3 genes, dN/dS = 0), microtubule-based movement (2 genes, dN/dS = 0), cytoskeleton organization (2 genes, dN/dS = 0), microtubule cytoskeleton organization (2 genes, dN/dS = 0), positive regulation of actin filament polymerization (2 genes, dN/dS = 0.01), cytokinesis (4 genes, dN/dS = 0.02), centriole replication (2 genes, dN/dS = 0.02), regulation of cytokinesis (2 genes, dN/dS = 0.02) and actin cytoskeleton organization (9 genes, dN/dS = 0.04). Much like the location annotation, a theme observed in the process annotation related to intracellular transport and included terms such as protein retention in ER lumen (2 genes, dN/dS = 0), calcium ion transport (3 genes, dN/dS = 0.01), golgi to endosome transport (2 genes, dN/dS = 0.01), vesicle docking involved in exocytosis (2 genes, dN/dS = 0.02) and retrograde transport, endosome to Golgi (3 genes, dN/dS = 0.03). Also present, was a protein synthesis/degradation theme supported by the terms proteasomal ubiquitin-dependent protein catabolic process (4 genes, dN/dS = 0.01), post-translational protein modification (7 genes, dN/dS = 0.02), RNA export from nucleus (3 genes, dN/dS = 0.02), protein ubiquination (10 genes, dN/dS = 0.03), translational elongation (15 genes, dN/dS = 0.03) and tRNA aminoacylation for protein translation (4 genes, dN/dS = 0.04). In contrast, process annotation terms exhibiting relatively large dN/dS ratios overlapped with themes of cellular signalling and regulation/response of cells to environmental signals.

Examples of process terms relating to these themes include neuroprotection (3 genes, dN/dS = 0.13), positive regulation of apoptosis (2 genes, dN/dS = 0.13), response to estradiol stimulus (6 genes, dN/dS = 0.14), response to peptide hormone stimulus (3 genes, dN/dS = 0.14), induction of apoptosis (10 genes, dN/dS = 0.15), positive regulation of MAPKKK cascade (2 genes, dN/dS = 0.16), cell surface receptor linked signalling pathway (5 genes, dN/dS = 0.16), response to antibiotic (4 genes, dN/dS = 0.18), response to hyperoxia (2 genes, dN/dS = 0.19), activation of caspase activity (5 genes, dN/dS = 0.21), response to cAMP (3 genes, dN/dS = 0.22), positive regulation of neuron apoptosis (3 genes, dN/dS = 0.24) and blood coagulation (6 genes, dN/dS = 0.22).

**Figure 5:**
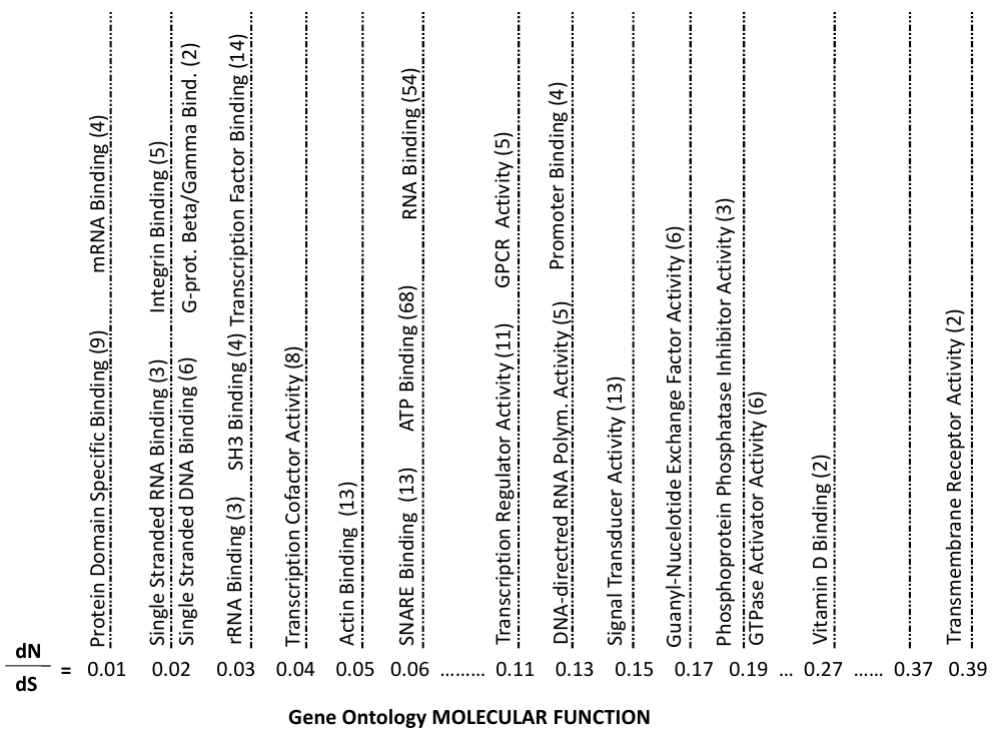
**Gene Ontology Molecular Function Terms by dN/dS Value**. Representative gene ontology molecular function terms associated with the proteins encoded by the feline cDNA sequences were stratified by dN/dS values of cat versus dog, human and mouse. The number of feline cDNAs associated with each annotation term is indicated in parentheses.

The gene ontology annotation for molecular function provides information about the structural and functional role of gene products. The overall theme within the low dN/dS group of function annotation involved molecules contributing to specific binding events (see Figure 5). Some of the annotation that supported this theme includes protein domain specific binding (9 genes, dN/dS = 0.01), mRNA binding (4 genes, dN/dS = 0.01), single stranded RNA binding (3 genes, dN/dS = 0.02), integrin binding (5 genes, dN/dS = 0.02), single stranded DNA binding (6 genes, dN/dS = 0.02), G-protein beta/gamma-subunit binding (2 genes, dN/dS = 0.02), rRNA binding (3 genes, dN/dS = 0.03), SH3 binding (4 genes, dN/dS = 0.03), transcription factor binding (14 genes, dN/dS = 0.03), transcription factor coactivator activity (8 genes, dN/dS = 0.04), actin binding (13 genes, dN/dS = 0.05), SNARE binding (4 genes, dN/dS = 0.06), ATP binding (68 genes, dN/dS = 0.06) and RNA binding (54 genes, dN/dS = 0.06). The theme associated with higher dN/dS associations with gene ontology molecular function annotation paralleled the theme observed in the high dN/dS biological process annotation, cellular response and signal transduction.

Function annotation terms associated with higher dN/dS values include transcription regulator activity (11 genes, dN/dS = 0.11), G-protein coupled receptor activity (5 genes, dN/dS = 0.11), DNA directed RNA polymerase activity (5 genes, dN/dS = 0.13), signal transducer activity (13 genes, dN/dS = 0.14), promoter binding (4 genes, dN/dS = 0.13), guanyl-nucleotide exchange factor activity (6 genes, dN/dS = 0.17), phosphoprotein phosphatase inhibitor activity (3 genes, dN/dS = 0.18), GTPase activator activity (6 genes, dN/dS = 0.19), vitamin D binding (2 genes, dN/dS = 0.26) and transmembrane receptor activity (2 genes, dN/dS = 0.39).

The themes observed in this data provide insight into the inner workings of the cell and shed light on the evolutionary constraints that act on different components of the intracellular machinery. The fact that the these feline sequences include a distribution of gene products, some of which are strongly conserved across human/mouse/dog, suggests that these sequences include genes that play very important roles in critical cellular processes and correspond to conserved mammalian cellular biology. However, some genes map to protein products that have relatively less selective pressure acting on them. These gene products are also important because they represent the targets of adaptive evolution within the cell. While microtubule structure and function must be highly conserved, regulatory gene products are freer to evolve new interactions that may increase fitness of the cell. Figures 3 through 5 contain the three types of gene ontology annotation together with the average dN/dS values for genes exhibiting the same annotation types. Although this analysis of dN/dS values across our genes provided a gene level picture of our data, we wanted to investigate the large-scale pattern of dN/dS values across our cDNA sequences.

### GeneGO Analysis of Orthologous Genes by dN/dS Value

In order to gain a more global view of how the feline cDNA sequences compared to other species, a set of 711 cDNA sequences having orthologs containing gene ontology annotation across dog, mouse and human were analysed to detect any non-random patterns across the genes, species and annotations. We sorted a list of 711 genes by dN/dS value and identified 3 groupings, corresponding to the top 25% of dN/dS values, the bottom 25% of dN/dS values and the middle 50% of dN/dS values. Each list was used to query the GeneGO annotation database for metabolic pathways.

**Figure 6:**
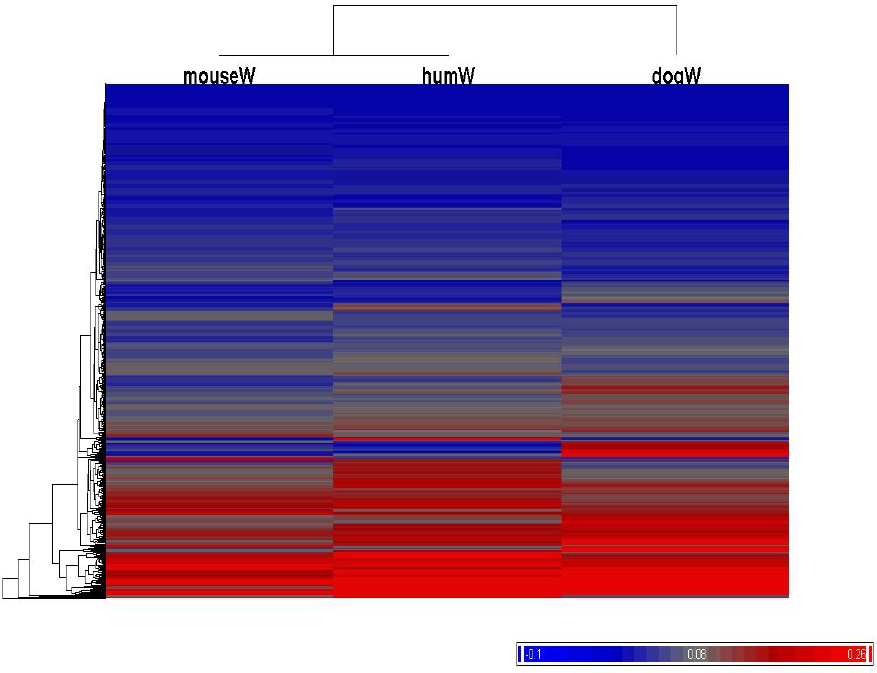
**Heat map of dN/dS values for Cat compared to Dog, Mouse and Human**. A set of 711 cDNA sequences with orthologs in dog, mouse and human were sorted by dN/dS (w) value to generate three groups corresponding to the top 25%, bottom 25% and middle 50%. Each list was used to query the GeneGO database for metabolic pathways. A non-random pattern was observed with genes with higher dN/dS (w) more frequently associated with metabolic pathways. Red indicates higher dN/dS (w) and blue corresponds to lower dN/dS (w) value.

The GeneGO database is based on the data and annotation of the Gene Ontology (GO) consortium which has collated biological annotations regarding the known or inferred roles of gene products, providing a powerful resource for identifying relationships among groups of genes, and thereby allowing the expansion of data analysis from single genes to gene sets. The GeneGO sowftare package identifies enrichment gene sets corresponding to metabolic and/or signalling networks using a hypergeometric model to calculate the null model probability for a set of genes. Enrichment is identified as an extremely unlikely probability under the null model. The results obtained by the GeneGO analysis indicate that the genes exhibiting higher dN/dS values were associated with specific metabolic pathways and biological processes. (Figure 6).

The heat map shows that for most genes, the dN/dS values are similar across different species. In order to see if any selection bias exists for different metabolic pathway annotations, the 711 genes were divided into 3 groups according to dN/dS value from dog/cat group. The first group contains the most conserved 178 genes with dN/dS values less than 0.0149, the second group contains the most divergent 178 genes with dN/dS values greater than 0.1229. The third group contains the remainder of genes having dN/dS values between 0.0149 and 0.1229 (see Table 5).

**Table 5:** Summary Statistics for GeneGO Annotation Analysis

	*All genes;* *All dN/dS*	*Top 25%;* *dN/dS < 0.0149*	*Middle 50%;* *0.0149 < dN/dS < 0.1229*	*Bottom 25%;* *dN/dS > 0.1229*	*Common networks between top 25% and bottom 25%*
**Genes**	711	178	355	178	

**Metabolic Networks**	91	29	79	42	12

**Network/Gene Ratio**	12.8%	16.3%	22.3%	23.6%	

A set of 711 cDNA sequences with orthologs across dog, mouse and human were stratified by dN/dS value into three groups corresponding to the top 25%, middle 50% and bottom 25% of dN/dS values. For each category, the number of sequences, and metabolic networks identified by GeneGO is shown. The number of metabolic networks shared between the groups representing the top and bottom quartiles is also shown.

We examined the metabolic networks of these genes in GeneGO. We observed that the group with lower dN/dS values exhibited fewer numbers of amino acid type metabolic networks than the group with larger dN/dS values. Our examination of these metabolic network annotations across the groups of genes provides insight into an interesting pattern that was not apparent from the gene level gene ontology analysis described in the preceding section.

We discovered that the group of genes with smaller dN/dS values are in metabolic networks exhibiting enrichment for carbohydrate metabolism, while the group with larger dN/dS values is associated with more metabolic networks involved in amino acid metabolism (See Table 6). Such patterns of more negative selection acting on carbohydrate metabolism and relatively less negative selection acting on amino acid metabolism may underlie an adaptive evolutionary role for genes associated with amino acid metabolism between obligate carnivores and omnivores. This result is in agreement with known differences in amino acid nutritional requirements between different species. This suggests that depending on dietary sources and metabolic requirements, the evolution rate may not be the same across all metabolic networks. These results provide an initial analysis of these genes and might be interpreted to suggest that genes associated with amino acid metabolism and biochemical utilization might have undergone different evolutionary selection among obligate carnivores compared to omnivores and herbivores. Such a hypothesis requires further exploration and may ultimately provide the genomic rationale of the need for feline specific nutritional needs that are distinct from other species, including dog.

**Table 6:** Conserved and Divergent Metabolic Pathways

*Most conserved*	*p-value*	*Most divergent*	*p-value*
1-icosatrienoyl-sn-glycero-3-phosphocholine pathway	6.01E-^03^	(L)-leucine pathways and transport	2.42E^-03^

1-docosahexaenoyl-glycerol_3-phosphocholine pathway	6.96E^-03^	GalNAcbeta1-3Gal pathway	3.06E^-03^

2-arachidonoyl-glycerol_3-phosphocholine pathway	8.16E^-03^	Branched-chain amino acid metabolism	4.08E^-03^

Phosphatidylinositol-3,4,5-triphosphate pathway	1.61E^-02^	Estrone and Estradiol metabolism	2.23E^-02^

[O-hexadecanoyl-(L)-carnitine pathway	2.10E^-02^	N-acyl-sphingosine phosphate pathway	2.48E^-02^

Phosphatidylinositol-4,5-diphosphate pathway	3.18E^-02^	HETE, HPETE and Leukotriene4 metabolism	6.47E^-02^

Glutamic acid pathway	3.83E^-02^	Tryptophan, Phenylalanine, Methionine metabolism	7.02E^-02^

L-glutamate pathways and transport	5.94E^-02^	Tryptophan, Phenylalanine, Tyramine, Methionine metabolism and transport	8.30E^-02^

Glycolysis, Glucogenesis and glucose transport	6.26E^-02^	Lyso-Phosphatidylserine pathway	8.88E^-02^

Glutamic acid pathways and transport	6.83E^-02^	Cholesterol biosynthesis	1.13E^-01^

The ten most highly conserved, and the ten most highly divergent metabolic pathways are listed along with the p-value for each pathway. The most conserved pathways are associated with the lowest dN/dS values, whereas the most divergent pathways are associated with the highest dN/dS values.

### Identification of Metabolic and Biochemical Pathways

Based on the GeneGO findings, we wanted to gain further insight into the biochemical role of the feline cDNA sequences. We chose to further explore how our cDNA sequences map onto metabolic pathways by identifying a set of pathways for which at least one pathway member has been identified in the set of our orthologous cat cDNA sequences. This analysis identified ten distinct classes of biochemical pathways for which 112 feline cDNA sequences have been mapped to 75 different pathways.

The categories of pathways include amino acid metabolism, biosynthesis of secondary metabolites, carbohydrate metabolism, energy metabolism, lipid metabolism, nucleotide metabolism as well as glycan biosynthesis and metabolism, metabolism of cofactors and vitamins and xenobiotic biodegradation and metabolism.

We identified 29 cDNA sequences in pathways underlying common amino acid metabolism pathways and 9 cDNA sequences involved in other amino acid metabolic pathways. We found 29 cDNA sequences that are involved in the metabolism of carbohydrates, 19 cDNA sequences involved in energy metabolism, 7 cDNA sequences associated with glycan biosynthesis and metabolism and 33 cDNA sequences that are involved in lipid metabolism. Additionally, we have identified 18 sequences that participate in the metabolism of cofactors and vitamins, 16 cDNA sequences that are involved in nucleotide metabolism and 12 that are involved in xenobiotic biodegradation and metabolism. Table 7 provides a summary of gene counts for these pathways.

**Table 7:** Genes Mapped to KEGG Pathways

*Pathway Category*	*Pathway Name*	*Number of Genes*
Amino Acid Metabolism	Alanine and aspartate metabolism	2
	
	Arginine and proline metabolism	3
	
	Glutamate metabolism	1
	
	Glycine, serine and threonine metabolism	3
	
	Histidine metabolism	2
	
	Lysine degradation	2
	
	Methionine metabolism	1
	
	Phenylalanine metabolism	3
	
	Phenylalanine, tyrosine and tryptophan biosynthesis	1
	
	Starch and sucrose metabolism	1
	
	Tryptophan metabolism	6
	
	Tyrosine metabolism	7
	
	Urea cycle and metabolism of amino groups	1
	
	Valine, leucine and isoleucine biosynthesis	2
	
	Valine, leucine and isoleucine degradation	3

Biosynthesis of Secondary Metabolites	Alkaloid biosynthesis II	1
	
	Limonene and pinene degradation	1

Carbohydrate Metabolism	Aminosugars metabolism	3
	
	Ascorbate and aldarate metabolism	1
	
	Butanoate metabolism	2
	
	Citrate cycle (TCA cycle)	3
	
	Fructose and mannose metabolism	2
	
	Galactose metabolism	1
	
	Glycolysis/Gluconeogenesis	6
	
	Glyoxylate and dicarboxylate metabolism	1
	
	Inositol phosphate metabolism	4
	
	Nucleotide sugars metabolism	1
	
	Pentose and glucuronate interconversions	1
	
	Pentose phosphate pathway	3
	
	Propanoate metabolism	1

Energy Metabolism	Methane metabolism	1
	
	Nitrogen metabolism	2
	
	Oxidative phosphorylation	15
	
	Sulfur metabolism	1

Glycan Biosynthesis and Metabolism	Glycan structures - biosynthesis 1	4
	
	Glycan structures - biosynthesis 2	4
	
	Glycosphingolipid biosynthesis - lacto and neolacto series	3
	
	Glycosylphosphatidylinositol(GPI)-anchor biosynthesis	1
	
	Heparan sulfate biosynthesis	1
	
	Keratan sulfate biosynthesis	2
	
	N-Glycan biosynthesis	2
	
	Peptidoglycan biosynthesis	1

Lipid Metabolism	alpha-Linolenic acid metabolism	1
	
	Androgen and estrogen metabolism	15
	
	Arachidonic acid metabolism	4
	
	Bile acid biosynthesis	1
	
	Biosynthesis of steroids	3
	
	Biosynthesis of unsaturated fatty acids	1
	
	Ether lipid metabolism	2
	
	Fatty acid elongation in mitochondria	1
	
	Fatty acid metabolism	4
	
	Glycerolipid metabolism	2
	
	Glycerophospholipid metabolism	6
	
	Linoleic acid metabolism	1

Metabolism of Cofactors and Vitamins	Biotin metabolism	1
	
	Folate biosynthesis	3
	
	Nicotinate and nicotinamide metabolism	2
	
	Pantothenate and CoA biosynthesis	3
	
	Porphyrin and chlorophyll metabolism	4
	
	Retinol metabolism	2
	
	Ubiquinone biosynthesis	2
	
	Vitamin B6 metabolism	1

Metabolism of Other Aminoacids	Aminophosphonate metabolism	1
	
	beta-Alanine metabolism	1
	
	Glutathione metabolism	7

Nucleotide Metabolism	Purine metabolism	14
	
	Pyrimidine metabolism	11

Xenobiotics Biodegradation and Metabolism	1- and 2-Methylnaphthalene degradation	2
	
	Benzoate degradation via CoA ligation	1
	
	Caprolactam degradation	1
	
	Drug metabolism - cytochrome P450	5
	
	Drug metabolism - other enzymes	3
	
	Geraniol degradation	1
	
	Metabolism of xenobiotics by cytochrome P450	5
	
	Styrene degradation	1

The human orthologs of feline cDNA sequences were used to identify biochemical and metabolic pathways in the KEGG database. A total of 112 cDNA sequences were mapped to 75 pathways. The table indicates the pathway category along with the pathway name and the number of genes mapped from each pathway.

### Comparative Phenotype Analysis

Phenotype annotation can provide additional information regarding the physiological function of a gene. Although our dataset includes 1227 cDNA sequences, one of our goals was to identify a relatively small subset of feline genes that represent important clinical, developmental and nutritional aspects of feline biology. This comparative phenotype analysis resulted in the identification of a pleiotropic set of genes that were partitioned into seven phenotype modules, each of which contains a relatively small number of genes that contribute to a comparatively large set of feline relevant phenotypes. The term phenotype module was adapted from the notion of a gene expression module, in which the set of genes exhibit similar patterns of spatial or temporal expression. Each phenotype module was constructed by grouping genes exhibiting related phenotypes based upon the phenotype classes described in the mammalian phenotype browser [40]. Similar phenotypes were grouped by body system and/or common biological processes to create the final set of phenotype modules. These seven modules provide a body system distributed view of the phenotypic roles of some of the genes that encode our 1227 cDNA sequences. The modules, genes and associated phenotypes are included in Table 8.

**Table 8:** Phenotype Modules and Feline Disorders

*Modules*	*Phenotypes*	*Disorders*
**Cardiac** 8 genes -Ras association (RalGDS/AF-6) domain family member 1 (cardiac hypertrophy [58,59]) -solute carrier family 22 (organic cation/carnitine transporter), member 5 (primary carnitine deficiency [75]) -cysteine-rich, angiogenic inducer, 61 (prostate cancer [76]) -tropomodulin 1 -transmembrane protein 38A -eukaryotic translation initiation factor 2-alpha kinase 1 -snail homolog 1 (Drosophila) (colon cancer [47]) -interleukin 1 receptor antagonist (coronary artery disease [77], autoinflammatory disease [54])	-cardiac hypertrophy -dilated dorsal aorta -abnormal mitral valve morphology -abnormal cardiac output -abnormal myocardial fiber physiology -enlarged heart -abnormal outflow tract -abnormal coronary artery morphology	-mitral valve dysplasia -tricuspid valve dysplasia -ventricular septal defects -atrial septal defects -dynamic subaortic stenosis -hypertrophic cardiomyopathy -restrictive cardiomyopathy -unclassified cardiomyopathy

**Development** 7 genes -TGFB-induced factor homeobox 2 -thioredoxin (Alzheimer's disease [78]) -E binding protein 1 -potassium inwardly-rectifying channel, subfamily J, member 1 (Andersen-Tawil syndrome [79], short QT syndrome [80]) -retinol dehydrogenase 12 (all-trans/9-cis/11-cis) (Leber's congenital amaurosis [81]) -arginine vasopressin receptor 1A -peptidylprolyl cis/trans isomerase, NIMA-interacting 1 (Alzheimer's disease [82])	-abnormal mesoderm development -abnormal proximal/distal developmental patterning -abnormal rostral/caudal developmental patterning -embryonic growth arrest -abnormal trophoblast layer morphology -abnormal white adipose tissue -decreased renal glomerular filtration rate -decreased cholesterol levels -decreased triglycerides, -abnormal intestine morphology -post natal growth retardation -retinal neuronal layer morphology -abnormal retinal apoptosis -abnormal circulating corticosterone level -abnormal adrenal gland morphology -decreased primordial germ cell number -seminiferous tubule degeneration	-tubular disease -chronic kidney disease -amyloidosis -membranous glomerulopathies -polycystic kidney disease

**Immune and Hematopoietic** 9 Genes -tumor protein D52-like 2 (childhood leukemia [83]) -fragile histidine triad gene (breast cancer [50], inflammatory bowel disease [84]) -tetraspanin 33 -beclin 1, autophagy related (Alzheimer's disease [51], colorectal cancer [56], glioblastoma multiforme [55]) -uracil-DNA glycosylase (hyper-IgM syndrome [57]) -solute carrier family 35, member C1 (leukocyte adhesion deficiency II [85]) -linker for activation of T cell -bridging integrator 3 -interleukin 1 receptor antagonist (coronary artery disease [77], autoinflammatory disease [54])	-abnormal macrophage physiology -abnormal hematopoiesis -abnormal T-cell proliferation -abnormal B-cell proliferation -increased susceptibility to infection -decreased granulocyte number -abnormal erythrocyte morphology -decreased hematocrit -decreased platelet number -abnormal immune system biology -increased spleen germinal cell number -increased spleen germinal size -abnormal class switch recombination -abnormal somatic hypermutation frequency -lymphoid hyperplasia -abnormal lymph node primary follicle -abnormal leukocyte adhesion -abnormal cytokine secretion -abnormal interferon secretion -increased IgE levels -increased IgG1 levels -increased IgM levels - increased interferon gamma secretion - increased interleukin 10 secretion - increased interleukin 4 secretion - enlarged spleen - liver inflammation - abnormal chemokine secretion - abnormal macrophage recruitment - increased susceptibility to endotoxin shock - chronic inflammation, - increased interleukin 1beta secretion - increased interleukin 17 secretion - increased interleukin 6 secretion	-Heinz bodies -pyruvate kinase deficiency -porphyria -lymphocytic cholangitis -neutrophilic cholangitis

**Energy, Nutrition and Homeostasis** 6 Genes -phosphatidylserine synthase 2 -glycerol kinase 2 -NADH dehydrogenase (ubiquinone) Fe-S protein 4, 18 kDa (NADH-coenzyme Q reductase) (Leigh syndrome [49]) -NAD(P)H dehydrogenase, quinone 1 (childhood acute lymphoblastic leukemia [41]) -solute carrier family 22 (organic cation/carnitine transporter), member 5 (primary carnitine deficiency [75])-syntaxin 4	-abnormal phospholipid level -increased circulating follicle stimulating hormone -decreased circulating glucose level -increased fatty acid level -abnormal body weight -decreased body temperature -decreased oxygen consumption -abnormal gluconeogenesis -abnormal liver morphology -decreased circulating glucose -increased glucagon -insulin resistance -abnormal glucose homeostasis -decreased circulating carnitine -hypoglycemia -increased circulating ammonia level -impaired glucose tolerance -insulin resistance -increased circulating insulin level	-insulin resistance -type II diabetes melitus Congts

**Tumorigenesis** 5 Genes -uracil-DNA glycosylase (hyper-IgM syndrome [57]) -caspase 9, apoptosis-related (colon cancer [42], inflammatory bowel disease [43]) -cysteine peptidase -bridging integrator 3 -beclin 1 (Alzheimer's disease [51], colorectal cancer [56], glioblastoma multiforme [55]) -Ras association (RalGDS/AF-6) domain family member 1 (cardiac hypertrophy [58,59])	-B-cell derived lymphoma -increased sensitivity to oxidative stress -increased apoptosis -decreased cellular sensitivity to gamma irradiation -increased incidence of ionizing radiation induced tumors -increased tumor incidence -malignant tumors -adenocarcinoma	-B-cell lymphoma -T-cell lymphoma -cutaneous mast cell tumors -visceral mast cell tumors -feline injection site sarcomas -squamous cell carcinoma -mammary gland tumors -meningioma

**Sensory** 5 Genes -bridging integrator 3 -mal, T-cell differentiation protein (protection against invasive pneumococcal disease, bacteremia, malaria and tuberculosis [45]) -NADH dehydrogenase (ubiquinone) Fe-S protein 4, 18 kDa (NADH-coenzyme Q reductase) (Leigh syndrome [49]) -caspase 9, apoptosis-related cysteine peptidase (colon cancer [42], inflammatory bowel disease [43]) -snail homolog 1(Drosophila) (colon cancer [47])	-abnormal lens fiber morphology -cataracts -abnormal optic nerve nerve morphology -abnormal eye electrophysiology -hyperkplexia -abnormal vision -blindness -optic nerve atrophy -decreased startle response -abnormal olfactory epithelium morphology - abnormal Meckel's cartilage morphology	-retinal degeneration -cataracts

**Behavior, Neurological and Nervous System** 12 Genes -N-ethylmaleimide-sensitive factor attachment protein, alpha -carbohydrate sulfotransferase 10 (melanoma [86]) -transmembrane protein 176B -solute carrier family 35, member C1 (leukocyte adhesion deficiency II [85]) -glycerol kinase 2 -NAD(P)H dehydrogenase, quinone 2 (Alzheimer's disease [87]) -NADH dehydrogenase (ubiquinone) Fe-S protein 4, 18 kDa (NADH-coenzyme Q reductase) (Leigh syndrome [49]) -diablo homolog (progressive hearing loss [88], breast cancer [89]) -caspase 9, apoptosis-related cysteine peptidase (colon cancer [42], inflammatory bowel disease [43]) -RAB3B, member RAS oncogene family -snail homolog 1(Drosophila) (colon cancer [47]) -mal, T-cell differentiation protein (protection against invasive pneumococcal disease, bacteremia, malaria and tuberculosis [45])	-abnormal motor coordination -impaired balance -impaired righting response -abnormal learning and memory -abnormal spatial learning, ataxia -abnormal maternal nurturing -abnormal posture -abnormal suckling behavior -hypoactivity -abnormal motor learning -abnormal spatial learning -abnormal spatial working memory -abnormal emotion and affect -abnormal gate -abnormal motor control -abnormal balance -abnormal vocalization -impaired coordination -abnormal nest building -abnormal stationary movement -abnormal CNS synaptic transmission -reduced long term potentiation -abnormal excitatory post synaptic potential -abnormal brain commissure morphology -abnormal brain development -abnormal embryonic neuroepithelium layer differentiation -decreased neuron apoptosis -decreased neurotransmitter release -enhanced paired-pulse facilitation -open neural tube -abnormal cerebellar granuale layer -abnormal Purkinje cell layer, small cerebellum -abnormal brain ventricle morphology -abnormal cerbral cortex morphology - abnormal forebrain morphology -abnormal hindbrain -abnormal myelination -abnormal neuron morphology -abnormal neuron physiology -astrocytosis, brain vacuoles -brainstem hemorrhage -gliosis -intercranial hemorrage	-lysosomal storage diseases -idiopathic vestibular disease -congenital unilateral vestibular disease

Mouse orthologs of feline cDNA sequences were used to identify phenotypes from the Mouse Genome Database. 38 genes were selected to represent seven phenotype modules. These genes were associated with a total of 136 phenotypes. Each of the seven modules is listed in the left column. The set of phenotypes associated with the genes in each module are indicated in the middle column. The clinically relevant feline disorders and diseases are listed in the third column. Gene names are listed in the left column. Specific human diseases that each gene is associated with are indicated in parentheses following the gene name and were identified by literature searches.

The cardiac module consists of eight genes and is associated with the following eight phenotypes: cardiac hypertrophy, dilated dorsal aorta, abnormal mitral valve morphology, abnormal cardiac output, abnormal myocardial fiber physiology, enlarged heart, abnormal outflow tract and abnormal coronary artery morphology. This module contains genes that are of relevance to feline cardiac disease such as hypertrophic cardiomyopathy and developmental defects of the heart.

The developmental-patterning module consists of seven genes and is associated with phenotypes that include abnormal mesoderm development, abnormal proximal/distal developmental patterning and abnormal rostral/caudal developmental patterning. Within this module we identified genes associated with distinct cell differentiation and specification properties such as embryonic growth arrest, abnormal trophoblast layer morphology and abnormal white adipose tissue. Additional phenotypes within this module were associated with retinal formation, renal function, intestine morphology as well as cholesterol, triglyceride and corticosterone levels. The phenotypes within this module may be useful in dissecting the genetic mechanisms underlying inherited developmental abnormalities in both domestic and endangered felids.

The third module is an immune and hematopoietic module that contains nine genes and represents phenotypes associated with specific cell types and lineages including macrophage physiology, spleen germinal cell number, granulocyte number, platelet number, T-cell and B-cell proliferation and hematopoiesis. Furthermore, this module exhibited phenotypes associated with susceptibility and resistance to pathogens such as abnormal immune system biology, abnormal class switch recombination and altered rate of infection. Some of the phenotypes in this module, including abnormal somatic hypermutation frequency and lymphoid hyperplasia were related to cancer, perhaps representing the immune surveillance component to the control of tumorigenesis within the body. Finally, some of the phenotypes within this module were associated with the modulation of specific immunologically important molecules such as cytokine secretion, interferon secretion, IgE levels, IgG1levels, and IgM levels. Genes within this module may offer some insight into feline specific immunological and inflammatory disorders.

The fourth module, energy/nutrition and homeostasis consists of six genes and exhibits a number of phenotypes associated with energy production and regulation within cells. Some of these phenotypes include decreased circulating glucose level, decreased oxygen consumption, abnormal gluconeogenesis and increased glucagon. Other phenotypes include endocrine level regulation of the organism such as abnormal body weight and decreased body temperature. Additionally, there were phenotypes associated with diseases of energy metabolism such as diabetes, these phenotypes included insulin resistance, abnormal glucose homeostasis and increased circulating insulin level. These phenotypes provide a context for better understanding of the unique nutritional and energy requirements of the cat.

The fifth module has five genes and encodes a tumorigenesis module associated with the following phenotypes: B-cell derived lymphoma, increased sensitivity to oxidative stress, increased apoptosis, decreased cellular sensitivity to gamma irradiation, increased incidence of ionizing radiation induced tumors, increased tumor incidence, malignant tumors, adenocarcinoma. The genes in this module may provide a useful gene set for investigating the genetic basis of feline lymphoma and carcinoma.

Module six is a sensory systems module, containing five genes, and is associated with the following visual phenotypes: abnormal lens fiber morphology, cataracts, abnormal optic nerve morphology, abnormal eye electrophysiology, abnormal vision, blindness and optic nerve atrophy. Cats exhibit vision related abnormalities under certain nutritional deficiencies; the genes associated with these phenotypes may provide a better understanding of the observed link between feline nutrition and visual function. Other phenotypes within this module include both hyperekplexia and decreased startle response, which may underlie feline adaptations required for successful predation.

The seventh module is a behavioral/neurological and nervous system set that contains 11 genes. The behavioral phenotypes arising from this module span traits as diverse as motor coordination and balance through learning, memory and gait. Additional phenotypes in this module are associated with emotion and affect as well as vocalization and maternal behavior. Within this module, we identified a number of phenotypes underlying neuronal specific physiological mechanisms such as altered synaptic transmission, altered long term potentiation, abnormal excitatory post synaptic potentials and decreased neurotransmitter release. This module contains a variety of developmentally important nervous system phenotypes having anatomical or histological annotations. These include abnormal brain commissure morphology, abnormal brain development, abnormal embryonic neuroepithelium layer differentiation as well as open neural tube, abnormal cerebellar granule layer, abnormal Purkinje cell layer, small cerebellum, abnormal brain ventricle morphology, abnormal cerebral cortex morphology and abnormal forebrain and hindbrain morphology. Finally, we identified specific CNS phenotypes of clinical importance such as abnormal neuron morphology, abnormal neuron physiology, astrocytosis, brain stem haemorrhage, gliosis and inter cranial haemorrhage.

We chose to focus on a relatively small number of gene-phenotype relationships in order to explore a relatively high resolution picture of important feline phenotypes that may be representative of our cDNA sequences. Our goal was to determine if any of our cDNA sequences were associated with phenotypes that may be of value in understanding the genetic basis of feline specific biology. Our analysis demonstrates that some of our cDNA sequences are indeed associated, through comparative genomics sequence analysis using the mammalian phenotype browser database, with phenotypes that are extremely important in feline health and disease. These modules and related genes provide an important and extremely useful candidate gene set for domestic cat functional genomics.

### Orthologous OMIM Diseases

We identified 104 feline cDNA sequences that are orthologs of human genes for which an OMIM (Online Mendelian Inheritance In Man, http://www.ncbi.nlm.nih.gov/omim) disease has been associated (see Table 9 and Additional file 3, Table S3). Within this data set we observe genes implicated in both dilated and familial cardiomyopathy as well as genes associated with oxidative phosphorylation deficiencies and biochemical disorders of amino acid metabolism. The OMIM associated diseases paralleled the phenotype associations we detected and provided additional insight into the clinical and nutritional role of the cDNA sequences we identified.

**Table 9:** List of OMIM Diseases

*Disease Name*	*Disease Name*
2-methyl-3-hydroxybutyryl-CoA dehydrogenase deficiency	Glycogen storage disease, type 0

2-methylbutyrylglycinuria	Gonadal dysgenesis, 46XY, partial, with minifascicular neuropathy

2-methylbutyrylglycinuria	Griscelli syndrome, type 2

3-methylglutaconic aciduria, type I	Hawkinsinuria

Acyl-CoA dehydrogenase, short-chain, deficiency of	Hemolytic anemia due to bisphosphoglycerate mutase deficiency

Adrenal cortical carcinoma	Homocysteine plasma level

Aldolase A deficiency	HPRT-related gout

Alzheimer disease-4	Hyper-IgD syndrome

Amyotrophic lateral sclerosis 10	Hyperleucinemia-isoleucinemia or hypervalinemia

Arthrogryposis multiplex congenita, distal, type 1	Hypervalinemia or hyperleucine-isoleucinemia

Bannayan-Riley-Ruvalcaba syndrome	Hypogonadotropic hypogonadism

Bartter syndrome, type 2	Hypokalemic periodic paralysis

Beta-ureidopropionase deficiency	Hypomagnesemia, renal, with ocular involvement

Birt-Hogg-Dube syndrome,	Hypotrichosis, localized, autosomal recessive

Bjornstad syndrome,	Immunodeficiency with hyper IgM, type 4

Breast cancer, sporadic	Leigh syndrome

Brugada syndrome 2	Leukoencephalopathy with vanishing white matter

Brunner syndrome	Lipoid adrenal hyperplasia

C2 deficiency	Lung cancer

C9 deficiency	Mast syndrome,

Cardiomyopathy, dilated, 1M,	Megakaryoblastic leukemia, acute

Cardiomyopathy, dilated, 1N	Mental retardation, X-linked syndromic

Cardiomyopathy, dilated, 1Z	Methemoglobinemia due to cytochrome b5 deficiency

Cardiomyopathy, familial hypertrophic	Methylmalonyl-CoA epimerase deficiency

Carnitine acetyltransferase deficiency	Microphthalmia, syndromic 6

Carnitine deficiency, systemic primary	Mitochondrial complex I deficiency

Cataract, posterior polar 2	Myopathy due to phosphoglycerate mutase deficiency

Cerebral dysgenesis, neuropathy, ichthyosis, and palmoplantar keratoderma syndrome	Myopathy with exercise intolerance, Swedish type

Ceroid lipofuscinosis, neuronal 8	Neuroblastoma

Charcot-Marie-Tooth disease, axonal, type 2F	Oral-facial-digital syndrome 1

Charcot-Marie-Tooth neuropathy, X-linked dominant, 1	Ovarian carcinoma

Colon cancer, advanced	Pancreatic cancer

Combined oxidative phosphorylation deficiency 2	Phenylketonuria - dihydropteridine reductase deficiency

Combined oxidative phosphorylation deficiency 5	Phosphoglycerate kinase 1 deficiency

Congenital disorder of glycosylation, type IIc	Porphyria cutanea tarda

Costello syndrome	Retinitis pigmentosa-46

Cutis laxa, autosomal dominant	Retinitis pigmentosa-46

D-2-hydroxyglutaric aciduria	Ribose 5-phosphate isomerase deficiency

Deafness, autosomal recessive 63	Spastic paraplegia 31

Desmosterolosis	Spondylocostal dysostosis, autosomal recessive 3

Diamond-Blackfan anemia 6	STAR syndrome

Epilepsy, neonatal myoclonic, with suppression-burst pattern	Temperature-sensitive apoptosis, cellular

Esophageal carcinoma, somatic	Transcobalamin II deficiency

Galactosemia	Tyrosinemia, type I

Generalized epilepsy with febrile seizures	Ventricular tachycardia, catecholaminergic polymorphic, 2

Glutamine deficiency, congenital	

A set of 90 human genetic disorders associated with orthologous feline cDNA sequences are listed in the table. The table contains an alphabetical list of the human diseases in two columns. (Additional information including cDNA identifier, ensembl human gene identifier, OMIM identifier and disease name can be found in Additional file 3, Table S3).

Within the set of OMIM diseases, we identified biochemical and metabolic diseases such as disorders of oxidative phosphorylation and glycosylation as well as D-2-hydroxyglutaric aciduria, glycogen storage disease, phenylketonuria due to dihydropteridine reductase deficiency and phosphoglycerate kinase 1 deficiency. Among the disease annotations associated with cancers, we found that our cDNA sequences were associated with specific types of OMIM annotations including breast cancer, colon cancer, esophageal carcinoma, lung cancer, pancreatic cancer and ovarian cancer to name a few. We also identified diseases of the sensory systems, such as cataracts and deafness. Finally, we discovered a variety of orthologs of human genes implicated in specific disorders, including Leigh syndrome, Hyper-IgD syndrome, immunodeficiency associated with hyper IgM, Griscelli syndrome, STAR syndrome and Charcot-Marie-Tooth disease, retinitis pigmentosa and generalized epilepsy. Together, these OMIM annotations provide a diverse picture of the genes across diseases and offer a unique context for understanding the role of these cDNA sequences in feline health and disease.

## Discussion

We identified 1227 feline cDNA sequences derived from tissues obtained from ten cats and performed extensive comparative genomics functional analysis to elucidate the computationally derived comparative gene expression analysis patterns, biochemical functions and phenotypes associated with these sequences. Our cDNA sequences and associated comparative and functional analysis provide an initial perspective on feline biology as viewed through our set of 1227 cDNA sequences. Although it is predicted that the number of feline protein coding genes encoded in the cat genome is in the order of 20,000 to 25,000, similar to most other mammalian genomes, the number of known published cat protein coding gene sequences is much lower at 2099 sequences (NCBI databse, 2011). These 1227 cDNA/gene sequences represent a rich set of potential targets for genetic association studies, biologically relevant diets and pharmacologically active compounds which can be developed to enhance the well-being of companion cats worldwide. Additionally, these sequences have value in similar applications for endangered felids.

Our strategy to identify a set of 1227 high quality and high confidence cDNA sequences from feline tissue samples expands the expressed sequence data for domestic cat. Although we initially obtained over 3000 cDNA sequences, we chose to filter our sequences so that the set we describe would be of the most value for the feline genomics community. Specifically, the conservative strategy outlined in Figure 1 resulted in a set of 913 known sequences and 314 novel sequences (1227 sequences in total) of which 914 orthologous clusters across feline, human, dog and mouse were identified (for which 844 were known cDNA sequences and 70 were novel cDNA sequences). The genes corresponding to these 914 orthologs were used as input sequences for a variety of bioinformatics and computational analyses aimed at providing an initial perspective on the physiological and pathological roles of these sequences in feline development, nutrition and health. Although we have identified a number of interesting results using computational and sequence comparison methods, our analysis only identifies the potential roles of these genes based on comparative analysis in other species. However, validating these results and proving the function of these genes will require molecular and biochemical experimental analysis. The results of our inferred expression analysis provide a set of gene expression patterns consistent with the source tissues used for cDNA production. Of the 21 source tissues used as starting material, inferred expression patterns from each anatomical region were detected with greater than 100 genes being associated in each case. It is interesting to note that each of these tissues exhibited relatively high gene expression numbers (i.e., numbers of genes associated with anatomical expression), which is what one would expect if the inferred expression patterns were an accurate representation of the true expression patterns of the source tissues. Tissues such as brain (725 genes), heart (629 genes), pancreas (568 genes) and testis (703 genes) exhibit inferred expression of more than 60% of the genes encoding our 1227 cDNA sequences. Inferred cellular expression patterns correlated with cell types expected in the source tissues including glial cells and neurons (432 genes and 124 genes respectively), retinal pigment epithelium cells (514 genes), and skeletal muscle cells (499 genes). Together, these results provide an expression framework for understanding the roles of these cDNA sequences in feline physiology and pathology. Because greater than 70% of our cDNA sequences were associated with embryological expression patterns we were not surprised to discover that a significant number of developmental phenotypes were associated with our set of cDNA sequences. Specifically, we identified genes associated with abnormal heart morphology and abnormal cardiac blood flow, abnormal mesoderm development, abnormal developmental patterning and abnormal retinal neuronal layer morphology. These phenotypes are consistent with the expression and role of genes identified in the source tissues selected for cDNA sequencing. The fact that the inferred expression patterns exhibit greater breadth of expression than the starting tissues is in line with the notion that genes tend to be expressed in complex spatial and temporal patterns. It may be the case that the inferred expression patterns include some anatomical, cellular and/or developmental expression patterns which may be false positives, however the overall picture of expression provided by this analysis greatly enhances the value of these cDNA sequences in genomic applications.

Interestingly, our analysis of gene ontology in the context of dN/dS values of individual orthologous cDNA sequences provides insight into how the domestic cat is both similar to and differs from other mammals. We detected evidence of negative selection acting on genes associated with microtubules and the actin cytoskeleton, suggesting that genes associated with these cellular structures are fairly well conserved among mammals [41,42]. Additionally, we identified gene ontology annotation terms affiliated with the nucleus, the chromosomes and DNA replication exhibiting relatively low values of dN/dS along with orthologs associated with transcriptional regulation and translational elongation. Similar values were obtained for genes annotated as G-protein beta/gamma binding and trans-Golgi network trafficking, vesicle and endoplasmic reticulum compartment membrane and SNARE complex. This is not surprising given that the housekeeping functions of mammalian cells are relatively well conserved. All cells must transmit information from the genome into RNA and protein components in a manner that maintains the appropriate subcellular compartmentalization of molecular functions. Intracellular trafficking that diverges from cellular requirements is likely to exhibit relatively deleterious consequences leading to negative selection compared to cells that function appropriately. Microtubules are involved in cellular integrity, cell motility and cell division; all of these processes are critical for cell viability [41,43].

In comparison to these highly conserved orthologs which mediate the core cellular processes, we detect evidence of considerably less negative selection acting on orthologs associated with transmembrane receptors, apoptotic signals, guanyl-nucleotide exchange factors and GPCR activity. Additionally, we identified evidence of less negative selection among orthologs associated with extracellular spaces, mitochondrial membrane affiliation and integral proteins of the plasma membrane. Unlike the highly conserved orthologs with intracellular functions, these orthologs form the basis of interactions across cells, through the extracellular space into the nucleus and organelles by a variety of signal transduction mechanisms for which multiple paralogous genes exist in each species. Such patterns of selection have been identified by others and represent evolutionary patterns of selection that may be associated with positive selection in different evolutionary lineages [44]. Moreover, these cDNA sequences might encode proteins for which extracellular environment plays a selective role during evolution.

It is well documented that paralogs diverge at a greater rate than orthologs [45,46]. Because our analysis did not include the entire set of genes from the cat, we cannot rule out the possibility that some of our orthologs are not true orthologs. It is worthwhile to point out that our analysis included only cat, dog, mouse and human genes which effectively limits the detection of evolutionary selection using the dN/dS ratio because some of these species diverged more than 100 million years ago. Nonetheless, it is interesting that others have observed similar patterns of divergence in protein networks operating at the cellular periphery and within the extracellular space [44,47].

Our analysis identified orthologs associated with respiratory chain and mitochondria as exhibiting relatively lower levels of negative selection. It is possible that the predatory status of cats resulted in adaptive changes in energy production and oxidative phosphorylation that facilitate the high energy requirements of predation.

It is interesting that we detect evidence of divergence within apoptotic genes in the cat compared to other mammalian species. This may underlie species specific differences in adaptation, such as what might be expected to have happened as obligate carnivores diverged from a common ancestor of omnivores and herbivores. The high protein requirements coupled with enhanced predatory fitness may have co-evolved with differences in cellular response to stress and cellular apoptosis, both within and outside of the brain.

This hypothesis is supported by the metabolic network analysis in GeneGO where the top 25% dN/dS values were associated with metabolic pathways implicated in non-carbohydrate roles. The metabolic network analysis performed with GeneGO demonstrated that genes in the group with smaller dN/dS values are associated with metabolic networks most involved in carbohydrate metabolism, while the genes in the larger dN/dS value group are in metabolic networks most involved in amino acid metabolism. This suggests that depending on metabolic requirements, the evolution rate may not be the same across all metabolic networks, and obligate carnivores like cats, may exhibit relatively less negative selection acting on genes involved in amino acid metabolism and more neutral selection acting on carbohydrate associated genes. This result is in agreement with the observation that cats exhibit different dietary requirements for amino acids taurine [20], arginine [21], cysteine and, methionine [22]. In contrast to dogs, cats are unable to synthesize taurine from cysteine [34], subsequently, taurine deficiency in cats is associated with a variety of clinically important conditions including cardiac [27] immune [28], neurological [29], platelet [30], reproductive [31] and retinal [32] dysfunctions. Additionally, cats exhibit rapid onset of ammonia toxicity resulting from arginine deficiency and, in severe cases, may die within 24 hours [23,48].

Through the use of KEGG pathway annotation, we identified domestic cat genes involved in a variety of amino acid related pathways including the metabolism of alanine, aspartate, arginine, proline, glutamate, glycine, serine, threonine, histidine, lysine, methionine, phenylalanine, tyrosine and tryptophan. We identified specific pathways in amino acid metabolism, which tend to differ between obligate carnivores and omnivorous mammals [49]. These include six genes involved in tryptophan metabolism which are of value for cats because they are unable to synthesize niacin from tryptophan, as compared to omnivores [48]. Additionally we identified three genes involved in arginine metabolism, which is an essential amino acid in cats [26]. We identified genes involved in glutamate metabolism, which may provide insight into the metabolic consequences of the low levels of ornithine produced from glutamate in cats [48].

We also identified genes associated with pathways underlying lipid metabolism, including genes participating in biochemical pathways of linoleic, alpha-linoleic acid and arachidonic acids, which is important and noteworthy because cats cannot use linoleic acid for the biosynthesis of arachidonic acid [48]. Further analysis of these genes may provide clues about feline biochemistry associated with arachidonic acid which may be important in feline reproduction [36]. Finally, we identified genes involved in the metabolism of retinol, which represent another very important gene set because cats are unable to synthesize retinol from beta-carotene [50].

The metabolism and biosynthesis of cofactors, vitamins and glycans is important in the nutrition and health of animals. Within these biochemical pathways, we identified three genes associated with folate metabolism, seven genes involved in glutathione metabolism and two genes associated with keratin sulfate biosynthesis, two genes associated with N-glycan biosynthesis and three genes associated with pathothenate and CoA biosynthesis. Some of these genes may provide value as important biological markers for monitoring oxidative stress, apoptosis and immune function in cats [51].

Collectively, many of these genes and their associated pathways are important for feline health and nutrition because they represent biochemical processes that cats have adapted to accommodate the narrow dietary range of an obligate carnivore in contrast to omnivorous mammals. The subsequent characterization of these genes and pathways may provide a genomic foundation for understanding how obligate carnivores differ from other animals in both health and disease.

Our functional and evolutionary analysis suggests that through divergent evolutionary trajectories, different species evolve slightly different biochemical processes of cells, tissues and organs that contribute to the manifestation of species specific adaptations and disorders. The domestic cat is known to suffer from a number of hereditary diseases, many of which have counterparts in other species like humans and dogs [52]. As part of our investigation into the biological significance of our cDNA sequences, we employed a comparative genomics approach to discover the phenotypes associated with these sequences. Our approach leveraged the mammalian phenotype ontology that has been developed as part of the mouse genome database [40]. We decided to select a relatively small number of genes for which a considerable number of important phenotypes may be associated.

Our phenotype data was obtained from previously published mouse phenotyping studies using transgenic or knockout mice. Subsequently, they should be considered as related to, rather than exactly, the true phenotypes that might arise in the cat. Because our method relies upon orthologous relationships between cat and mouse genes, it is worthwhile to point out that inaccurate mappings between orthologs may lead to inaccurate predictions of phenotypes. Furthermore, as we have described throughout this paper, the cat exhibits some strong similarities to general biological processes that are shared with mammals. The cat also has well documented differences when compared to omnivorous animals. Therefore, one must consider the phenotype analysis as a general thematic picture of the functional consequences of our cDNA sequences rather than as a one-to-one mapping of gene-phenotype associations within our cDNA sequences.

We identified seven phenotypic modules exhibiting 136 phenotypes arising from only 38 genes. Many of the genes we identified exhibit numerous phenotypes, both within and across modules. Such pleiotropic effects underlie the complexity of mammalian genomes and provide context for future genomic studies. We selected these gene-phenotype associations to provide a detailed, but yet tractable picture of how our cDNA sequences might map onto anatomical and physiological traits.

Within the cardiac module, we identified eight genes associated with phenotypes relating to cardiac disease in cats. Some of the genes within this module include tropomodulin 1, snail homolog 1 and an interleukin receptor antagonist. This module includes phenotypes of cardiac hypertrophy and mitral valve defects, both of which are known hereditary diseases in cats [53]. These genes provide examples of the types of phenotypes that might arise from perturbations of cat genes underlying inherited feline cardiac diseases, such as aortic stenosis, atrial-septal defect, mitral valve displasia, tetralogy of Fallot and ventricular-septal defect [53,54].

Our developmental module consists of seven genes and includes a TGFbeta induced homeobox transcription factor as well as the signaling molecule arginine-vasopressin. The phenotypes associated with this module include developmental patterning across both the proximal/distal axis and the rostral/caudal axis. The phenotypes also include cellular specification and patterning such as mesoderm development, trophoblast layer morphology and adipose tissue differentiation, to name a few. Domestic cats exhibit a variety of developmental defects, such as polydactyly, hip dysplasia, sacrococcygeal dysgenesis, portocaval shunt, open central fontanel, open lateral fontanel and thoracic hemivertebra [54-57]. The cDNA sequences we describe may include genes that are responsible for abnormal developmental conditions in domestic and endangered felids.

We identified a sensory module, which contains five genes such as NADH dehydrogenase (ubiuinone) Fe-S protein 4 and caspase 9 apoptosis-related cysteine pepidase. This module includes the phenotypes of cataracts, blindness and optic nerve atrophy. Examples of inherited sensory system disorders in the domestic cat include cataracts, corneal dystrophy (stromal and endothelial), progressive retinal atrophy and glaucoma [58]. The overlap between retinal and ocular phenotypes and inherited feline diseases suggests that there are specific genomic regions, represented by our cDNA sequences, which may include aspects of the genetic mechanisms of these debilitating diseases in cats. It is interesting to note that our sensory module includes genes involved in energy production. This is not surprising as retinal tissue is known to exhibit relatively high energy requirements and depletion of energy in this tissue has been associated with blindness and other vision defects [50].

Within our energy and homeostasis module, we identified genes like glycerol kinase 2, NAD(P)H dehydrogenase quinone 1 and NADH dehydrogenase (ubiquinone) Fe-S protein 4. The phenotypes within this module are associated with traits of clinical and adaptive importance in the cat. For example, our comparative phenotype analysis identified phenotypes of insulin resistance, increased circulating insulin level and impaired glucose tolerance; traits associated with the feline hereditary disease of diabetes mellitus [59]. This module also contains phenotypes such as abnormal gluconeogenesis, increased glucagon, abnormal glucose homeostasis and increased circulating ammonia level, which are important in felid nutrition as cats use gluconeogenesis as a predominant form of energy production and are susceptible to ammonia toxicity [17,18]. The genes in this module are of value in exploring some of the fundamental metabolic and biochemical differences between obligate carnivores and omnivores. Moreover, these genes may provide a genomic basis for specific diets that can reduce the incidence of feline disorders associated with specific nutritional deficiencies.

Within other modules, we identified phenotypes associated with cancer, such as increased tumor incidence, malignant tumors and B-cell derived lymphoma which may provide clues to the genetic susceptibility cats have for hereditary lymphoma [60]. Among the behavioral phenotypes within the nervous system module, we identified a number of traits that may represent predator specific adaptations of cats. For example, we identified cDNA sequences associated with spatial learning, balance, righting response, gate and motor coordination; traits that are almost synonymous with cats and of extreme adaptive value for an apex hyper predator.

The comparative genomics analysis of OMIM diseases within our cDNA sequence data set provides a final perspective on the importance of our reported sequences in the health of domestic cats. Many of the diseases identified in the OMIM mapping are also represented by phenotypes within the modules. This independent annotation demonstrates that our analysis converges even though OMIM analysis leverages human orthology relationships and the phenotype analysis leverages murine orthology relationships. It is worth noting the limitation of sequence based comparative genomics approaches. They can provide considerable insight into the functional role of our cDNA sequences, but must ultimately be proven through focused and carefully designed genomics studies in cats. Nonetheless, our cDNA sequences and associated analysis provide considerable value through the identification of many interesting clinically and nutritionally relevant feline genes.

The set of diseases and phenotypes provides a starting point for candidate gene approaches and for the selection of biomarkers for monitoring nutrition and health. By combining diverse types of annotation, we can better understand the function of a given gene in a breadth of tissues and organ systems and of the biological processes it is involved in the organismal level, as well as its role in disease. For example, we identified genes associated with expression in the heart, and with a number of cardiac phenotypes, including cardiac hypertrophy, abnormal outflow tract and abnormal mitral valve morphology, as well as the OMIM disease annotation of dilated cardiomyopathy. These are of direct relevance to feline disease, since hypertrophic cardiomyopathy is a common clinical concern in cats [53].

The recent development of a 70,000 SNP feline bead array by Hill's Pet Nutrition and the Morris Animal Foundation provides an important and powerful resource for conducting gene association studies in the domestic cat, and related endangered species. However, even in the absence of whole-genome genetic association approaches, our characterization of these 1227 cDNA sequences provides an extremely valuable resource for candidate gene approaches aimed at investigating the genetic basis of feline phenotypes. It will be interesting to see how our comparative and functional analysis of these 1227 cDNA sequences compares to the data produced from high throughput sequencing and future genetic studies within and across different breeds in the domestic cat. It is likely that some of our functional annotations may turn out not to hold, and it is equally likely that some of them will. Through collaborative efforts, it will be possible to begin unravelling the genetic mechanisms underlying feline health and disease.

## Conclusions

We report the identification of 1227 feline cDNA sequences of which, 913 correspond to higher quality versions of public feline sequences and 314 correspond to novel feline sequences for which no known public sequence data exists. Our comprehensive functional analysis identified a number of physiologically important biochemical pathways that these sequences are involved in as well as of the developmental, clinical and nutritional relevant phenotypes they are associated with.

## Methods

### Construction of feline tissue specific cDNA libraries

The study protocol was reviewed and approved by the Institutional Animal Care and Use Committee. All cats were immunized against feline panleukopenia, calici, rhinotracheitis, and rabies. Cats were housed with 10 - 12 other cats and food was continuously available throughout the day until their daily caloric requirements were consumed. Cats were housed in spacious rooms with natural light that varies with seasonal changes. Cats experienced behavioral enrichment through interactions with each other, by daily interaction and play time with caretakers, large windows and sun porches to watch the natural landscape and access to toys. At the end of their natural life, cats were euthanized for humane purposes and tissues were stored at -80C.

Total RNA was purified from 21 feline tissues (brain, kidney medulla/cortex, spleen, heart, liver, lung, skeletal muscle, thyroid gland, lymph node, pancreas, adrenal gland, tongue, colon, mammary gland, neonatal thymus, brain and testes) collected from 10 domestic short-haired cats postmortem, three cell lines derived from kidney, brain, lung, and 1 tissue pool using standard procedures as described in [48]. The purity and integrity of each RNA sample was assessed by spectrophotometry and gel electrophoresis. Forty normalized cDNA libraries were constructed by Agencourt Inc. (Beckman-Coulter Genomics), 22 with standard inserts (1.2 kb) and 18 with long inserts (> 4 kb). The first and second cDNA strands were synthesized using optimized methods, and cDNAs were size selected prior to cloning. The size-selected cDNAs were directionally cloned into the pAGEN vector by polishing and restriction digest, creating a 5' blunt end and a 3' overhang.

Each cDNA library was subsequently tested for specific quality control measures (average insert size, number of independent clones and percentage of recombinant clones), and normalized to reduce the proportion of highly abundant mRNAs. Normalization was performed by dividing each library into two populations, using the first for *in vitro *transcription of biotinylated RNA, and the second to generate single stranded phagemid DNA. The two populations were then mixed, and self-hybridized DNA-RNA molecules corresponding to over-represented mRNAs were removed. The remaining single stranded DNA molecules were primed for second strand synthesis and the resulting clones were transformed into bacteria, yielding the normalized libraries.

### Sequencing of feline cDNA libraries

Plasmids were purified from each library using a large-scale automated protocol, the SprintPrep^® ^Solid Phase Reversible Immobilization procedure. Sequencing reactions were performed in 384-well plates using BigDye^® ^Version 3.1 direct cycle sequencing (Applied Biosystems, CA). Sequencing reactions were purified using the CleanSeq^® ^dye-terminator removal kit (Agencourt, Inc.), and resolved by capillary electrophoresis using the ABI3730 Genetic Analyzer (Applied Biosystems, CA). Sequencing reads were processed using Phred and quality scores for each run were monitored using the Agencourt, Inc. Galaxy LIMS system. Sequencing of these cDNA libraries yielded a total of 919,676 EST reads.

### Data Management and Analysis

The sequence data, annotation data and the data resulting from sequence analysis were loaded into the MySQL relational database version 5 to facilitate data management and analysis [61].

### Sequence Filtering and Ortholog Detection

A set of 3035 full length feline cDNA sequences were obtained from the analysis of the sequencing data and used to identify a set of high confidence cDNA sequences. All cDNA sequences were translated in 6 reading frames and the longest protein coding sequence obtained was noted. These cDNA and protein sequences were clustered using blast to identify a set of non-redundant nucleotide and non-redundant protein sequences using a stringency of 95% or greater as criteria for identifying redundant sequences. For each cluster, the longest representative sequence was chosen as the non-redundant representative. The intersection of non-redundant nucleotide sequences and non-redundant protein sequences was used as the set of non-redundant sequences.

The BLAST programs, blastp and blastn [62,63], were run with the non-redundant full length feline sequences as query and the target species sequences downloaded from ENSEMBL ftp://ftp.ensembl.org/pub/current/fasta/[36] as subject sequences. The subject sequences for each of the four species (dog, cat, human and mouse) were: Homo_sapiens.GRCh37.60.cdna.all.fa (containing 147,141 sequences), Homo_sapiens.GRCh37.60.pep.all.fa (containing 81,968 sequences), Mus_musculus.NCBIM37.60.cdna.all.fa (containing 82,508 sequences), Mus_musculus.NCBIM37.60.pep.all.fa (containing 50,959 sequences),

Canis_familiaris.BROADD2.60.cdna.all.fa (containing 27,301 sequences),

Canis_familiaris.BROADD2.60.pep.all.fa (containing 25,559 sequences),

Felis_catus.CAT.60.cdna.all.fa (containing16,332 sequences) and

Felis_catus.CAT.60.pep.all.fa (containing 15,048 sequences). Because the human sequence sets contain the greatest number of target sequences, 147,141 nucleotide sequences and 81,968 protein sequences, the set of non-redundant sequences were mapped to the human sequences. Additionally, the full length sequences were mapped to the set of known feline cDNA and protein sequences in order to classify the full length non-redundant feline sequences as either *known *or *novel*, where *known *indicates that the sequence is represented by a feline sequence in the public ensembl transcript/protein sequence data while *novel *indicates that the sequence does not have a representative transcript or protein sequence in the ensembl data set.

Because the public feline data does not contain all of the protein coding genes, it was not possible to perform an ortholog search using the standard reciprocal best hit approach. Instead, the blast results were filtered using an iterative heuristic process of selecting blast hits with specific match lengths, gaps, number mismatches and percent identity. In total, eight iterative steps were performed beginning with the most stringent and ending with the least stringent. Each step identified a set of qualifying non-redundant full length sequences. The first and most stringent step imposed the requirement that the blast match_length must be equal to the smallest of the two sequences (query or subject) and the number of mismatches = 0, number of gaps = 0, and the percent identity ≥ 99%. A second filter was used to add additional sequences to the results of the first step, and any sequences that had not been identified in the first step were added to the set of results. The second step used a blast match_length ratio of ≥ 0.99, number mismatches = 0, number gaps = 0 and percent identity ≥ 99%. A third step identified additional sequences that satisfied the third step criteria and for which the first two steps did select the non-redundant full length sequence. The third step criteria were blast match length ratio ≥ 0.87, number of mismatches ≤ 4, number of gaps = 0, and percent identity ≥ 99%. The iterative process continued for a total of eight steps with each subsequent step relaxing the filtering criteria in order to identify sequences that were not identified in the previous step. Fourth step criteria were blast match length ratio ≥ 0.725, number mismatches ≤ 5, number of gaps = 0, and percent identity ≥ 99%. Fifth step criteria were blast match length ≥ 0.69, number mismatches ≤ 4, number gaps ≤ 1 and percent identity ≥ 99%. Sixth step criteria included blast match length ≥ 0.625, number mismatches ≤ 8, number of gaps ≤ 1, and percent identity ≥ 98%. Seventh step criteria included blast match length ratio ≥ 0.575, number mismatches ≤ 13, number gaps ≤ 2 and percent identity ≥ 97%. The eighth step criteria were blast match length ≥ 0.52, number mismatches ≤ 12, number of gaps ≤ 2 and percent identity ≥ 97%.

The resulting set of non-redundant full length sequences were considered to represent the high quality feline cDNA and protein sequences. These high quality sequences which mapped to a *known *public feline sequence were used to generate global nucleotide and protein alignments using the partial order alignment software POA http://bioinfo.mbi.ucla.edu/poa2/POA_Online/Align.html[64,65]. All alignments were manually inspected to ensure that each non-redundant full length feline sequence mapped to the correct public feline sequence.

### Comparative Expression Analysis

In order to infer anatomical and cellular expression patterns of our sequences, four expression annotation files were downloaded from the public biomart http://www.biomart.org[66] web server. Because we mapped our sequences to their corresponding human orthologs, we downloaded the human biomart egenetics annotation data sets mapped on top of the ensembl gene 60 version human gene identifiers. The four annotation sets we obtained included human ensembl gene identifiers mapped to (1) a set of anatomical terms, (2) a set of cell types, (3) a list of pathological terms and (4) a list of developmental stages ranging from weeks to years.

Although our sequences represent a subset of gene products, we found value in identifying the spectrum of expression patterns these sequences may exhibit beyond the tissue libraries that we used. The mapping was accomplished by loading each of the four gene expression annotation files into the MySQL relational database and performing SQL queries that joined these expression tables to our orthologous gene set using the ensembl human gene identifier.

### dN/dS Codon Substitution Rate Calculations

In order to better understand the evolutionary relationships between the feline cDNA sequences and the orthologous sequences in dog, human and mouse, we calculated dN/dS values for orthologous sequences across the different species. Phylogenetic Analysis by Maximum Likelihood (PAML version 4.4) software was used to run the codon stats using the "codeml" program. Codon stats were computed where it was possible (DNA and protein sequence availability for the orthologs) with basic model (NSSites = 0) ω = *dN/dS*, the ratio of nonsynonymous/synonymous substitution rates

The ω ratio is a measure of natural selection acting on the protein. Simplistically, values of ω < 1, = 1, and > 1 means negative purifying selection, neutral evolution, and positive selection respectively. PAL 2NAL [67-69] was used to create codon alignments between the cDNAs and the proteins to input to PAML program which computes the dN, dS and ω ratio. Codon substitution rate data was loaded into the MySQL relational database and used to assess the evolutionary pressure exerted on specific groups of genes. The gene groups were derived from other annotation types, such as gene ontology and phenotype annotation results.

### Gene Ontology Annotation

Gene ontology annotation was added to the set of orthologous sequences via comparative sequence analysis. Because the orthologous sequences were already mapped to the human transcripts and proteins, we decided to download the gene ontology annotation files corresponding to biological process, molecular function and cellular localization in order to annotate the non-redundant full length feline sequences with gene ontology terms [70]. The gene ontology annotation files linking the gene ontology terms to the ensembl human gene identifiers were obtained from biomart http://www.biomart.org. Each feline sequence we identified was annotated with all of the gene ontology terms associated with the orthologous human gene. In this manner, we were able to identify a larger set of gene ontology annotations per feline gene than we could have accomplished if we limited the annotation mapping to only the feline cDNA sequences we identified. Through this greedy algorithm, we were able to gain a more comprehensive understanding of the genes we identified. SQL queries in MySQL database were used to map the human gene ontology annotation terms to the orthologous feline genes encoding the cDNA sequences we identified.

### GeneGO Metabolic Network Analysis

Metabolic networks of feline sequences (using ENSEMBL id to upload) were performed using the MetaCore software (GeneGO, St. Joseph, MI). MetaCore identifies networks based on a manually curated database containing known molecular interactions, functions, and disease interrelationships. The networks are identified by the probability that a random set of genes the same size as the input list would give rise to a particular mapping by chance. Therefore, an enrichment of biological relevant pathways or networks can be found.

### KEGG Pathway Annotation

Pathway associations were identified using a comparative genomics approach. Because our orthologous sequences were mapped to human orthologs, it was possible to use the human pathway association information to map the pathways on the orthologous feline sequences. This was accomplished using SQL queries to join the KEGG http://www.genome.jp/kegg/pathway.html[71] and Biocarta http://www.biocarta.com/genes/index.asp pathway data [72] that has been associated with human ensembl gene identifiers with feline gene identifiers. Additional pathways were identified using the David Bioinformatics Database http://david.abcc.ncifcrf.gov/[73] through a gene set search using the ensembl gene identifiers for the set of human orthologs of the feline sequences we identified.

### Comparative Phenotype Mapping

Gene specific phenotype annotation derived from mouse knockouts and/or transgenic strains is compiled and made publicly available at the Mouse Genome Database http://www.informatics.jax.org/[40]. The phenotype annotation is structured within the mammalian phenotype ontology which provides an acyclic graph of mammalian morphological and physiological phenotypes. Because the mouse phenotype data is associated with each mouse gene, it was possible to link the mammalian phenotype ontology to the feline non-redundant full length sequences through a two step process. First, the mammalian phenotype annotations linked to mouse gene identifiers were obtained and loaded into the MySQL database. Next, the appropriate SQL query was performed which created a table that joined the phenotype information with our feline sequence data. The resulting phenotype annotations on top of the feline orthologous gene set provide an additional mechanism for understanding the role of these cDNA sequences in cat development, health and disease.

### OMIM Disease Mapping

A comparative genomics map of our feline sequences annotated with the OMIM http://www.ncbi.nlm.nih.gov/omim[74] disease information was generated using two different approaches. The first approach utilized MIM disease data that was produced from biomart and anchored to the human ensembl gene identifiers. The resulting annotation file was loaded into the relational database and an appropriate SQL query was used to connect the disease information to the feline sequences through the orthologous relationships that were previously determined. The resulting mapping provided formal associations between feline cDNA sequences and OMIM disease information http://www.ncbi.nlm.nih.gov/omim.

A second method of mapping the feline sequence data to the OMIM data was used to improve the set of OMIM annotated feline cDNA sequences. Specifically, the set of human ensembl gene identifiers corresponding to the orthologs for the feline cDNA sequences were used to query the David Bioinformatics database for OMIM disease information. The resulting file downloaded from the David Database contained human ensembl gene identifiers and OMIM disease identifiers. This file was loaded in the MySQL database and linked with the non-redundant feline cDNA sequences using an appropriate SQL query.

## Competing interests

Western University of Health Sciences received funding from Hill's Pet Nutrition to support KJI's time on this project.

## Authors' contributions

All authors have read and approved the final manuscript.

KJI co-developed the experimental strategy, performed comparative genomics analysis of cDNA sequences and wrote the manuscript. SBM performed dN/dS calculations, managed orthologous data sets, provided critical review of results and contributed to manuscript. XG performed GeneGO analysis, provided critical review of results and contributed to manuscript. KM provided interpretation and relevance of comparative genomics annotation data sets and contributed to manuscript. LM provided interpretation and clinical relevance of comparative genomics annotation data sets and contributed to manuscript. PB provided interpretation and clinical relevance of comparative genomics annotation data sets and contributed to manuscript. JAB responsible for the cDNA sequencing data, provided critical review of experimental approach and contributed to manuscript. SWA co-developed the experimental strategy, responsible for the cDNA sequencing data, managed the project and coordinated data production and manuscript preparation.

## Supplementary Material

Additional file 1 **The cDNA and protein sequences and other information corresponding to the 1227 identified feline sequences**. This table lists both the cDNA and protein sequences and corresponding lengths for each of the 1227 feline sequences we identified, along with the designation novel (314 sequences) or known (914 sequences). Sequence Identifier (unique identifier for each of the 1227 non-redundant feline sequences). Status (*known *denotes sequences that map to public feline sequences and *novel *denotes sequences with no identity to public feline sequences). cDNA Sequence Length (nucleotide length of cDNA sequence). cDNA Sequence (nucleotide sequence of cDNA). Protein Sequence Length (amino acid length of protein sequence). Protein Sequence (protein sequence corresponding to longest translation product of the cDNA). Click here for file

Additional file 2 **Sequences and other information on orthologous sequences from the dog, human and mouse**. This table contains the 914 orthologous sequences (844 known and 70 novel) and the corresponding ensembl gene, transcript and protein identifiers for the dog, human and mouse orthologs. Status (*known *denotes sequences that map to public feline sequences and *novel *denotes sequences with no identity to public feline sequences). Symbol (gene symbol). Title (gene title). Sequence Identifier (unique identifier for each non-redundant feline sequence). Cat Gene Id (cat ensembl gene identifier for sequences designated as *known*). Cat Transcript Id (cat ensembl transcript identifier for sequences designated as *known*). Cat Protein Id (cat ensembl protein identifier for subset of sequences designated as *known*). Dog Gene Id (ensembl gene identifier of dog ortholog). Dog Transcript Id (ensembl transcript identifier of dog ortholog). Dog Protein Id (ensembl protein identifier of dog ortholog). Human Gene Id (ensembl gene identifier of human ortholog). Human Transcript Id (ensembl transcript identifier of human ortholog). Human Protein Id (ensembl protein identifier of human ortholog). Mouse Gene Id (ensembl gene identifier of mouse ortholog). Mouse Transcript Id (ensembl transcript identifier of mouse ortholog). Mouse Protein Id (ensembl protein identifier of mouse ortholog). Click here for file

Additional file 3 **Feline Genes Mapped to OMIM Diseases**. This table contains a set of 104 feline cDNA sequences that were mapped to their corresponding human orthologs and the associated OMIM diseases. The first column indicates the cDNA identifier, the second column contains the ensembl human gene identifier for the orthologous human gene, and the third and fourth columns contain the OMIM disease identifier and the disease name respectively. Sequence Identifier (unique identifier for each non-redundant feline sequence). Human Gene Id (ensembl gene identifier of human ortholog). OMIM Identifier (OMIM id for a specific human disease). Disease Name (the name of the disease from the OMIM database). Click here for file
